# Exercise Challenge in Gulf War Illness Reveals Two Subgroups with Altered Brain Structure and Function

**DOI:** 10.1371/journal.pone.0063903

**Published:** 2013-06-14

**Authors:** Rakib U. Rayhan, Benson W. Stevens, Megna P. Raksit, Joshua A. Ripple, Christian R. Timbol, Oluwatoyin Adewuyi, John W. VanMeter, James N. Baraniuk

**Affiliations:** 1 Division of Rheumatology, Immunology and Allergy, Department of Medicine, Georgetown University Medical Center, Washington, District of Columbia, United States of America; 2 Department of Neurology, Center for Functional and Molecular Imaging, Georgetown University Medical Center, Washington, District of Columbia, United States of America; 3 Cognitive Neurogenetics Laboratory, Department of Psychology, Georgetown University, Washington, District of Columbia, United States of America; Cuban Neuroscience Center, Cuba

## Abstract

Nearly 30% of the approximately 700,000 military personnel who served in Operation Desert Storm (1990–1991) have developed Gulf War Illness, a condition that presents with symptoms such as cognitive impairment, autonomic dysfunction, debilitating fatigue and chronic widespread pain that implicate the central nervous system. A hallmark complaint of subjects with Gulf War Illness is post-exertional malaise; defined as an exacerbation of symptoms following physical and/or mental effort. To study the causal relationship between exercise, the brain, and changes in symptoms, 28 Gulf War veterans and 10 controls completed an fMRI scan before and after two exercise stress tests to investigate serial changes in pain, autonomic function, and working memory. Exercise induced two clinical Gulf War Illness subgroups. One subgroup presented with orthostatic tachycardia (*n = *10). This phenotype correlated with brainstem atrophy, baseline working memory compensation in the cerebellar vermis, and subsequent loss of compensation after exercise. The other subgroup developed exercise induced hyperalgesia (*n = *18) that was associated with cortical atrophy and baseline working memory compensation in the basal ganglia. Alterations in cognition, brain structure, and symptoms were absent in controls. Our novel findings may provide an understanding of the relationship between the brain and post-exertional malaise in Gulf War Illness.

## Introduction

Gulf War Illness (GWI) has affected 25% to 30% of the approximately 700,000 military personnel who served in the 1990–1991 Persian Gulf War [1]. Veterans present with multifaceted symptom profiles that include cognitive impairment, widespread pain, interoceptive complaints and autonomic dysfunction [2]–[4]. There are no validated clinical markers for GWI to account for inter-individual variations in symptom severity or differences from controls. Ambiguity is increased by the use of multiple epidemiologically derived criteria and non-standardized symptom assessments [1], [3]–[5]. Research suggests a prominent neurological component, but no unifying disease mechanisms have emerged [6]–[11]. GWI shares subjective symptoms with other idiopathic illnesses that include chronic fatigue syndrome (CFS) and fibromyalgia [5], [12]. Similar to CFS and fibromyalgia, GWI subjects complain of exertional malaise with severe exacerbations of baseline symptoms following a physiological stressor [12]–[15]. Exercise is a useful model to study symptom alterations in CFS, fibromyalgia and GWI [15]–[17]. However, the causal relationships between exercise, the brain, and deteriorating disease status are unknown.

We hypothesized that acute physiological stressors would exacerbate symptoms and identify the predominant mechanisms associated with central nervous system dysfunction. The effects of 2 bicycle exercise stress tests performed on consecutive days on widespread pain (hyperalgesia), autonomic regulation, and working memory function were studied in 10 controls and 28 Gulf War veterans who met the 1998 CDC case definition criteria for GWI over a four day period [4]. Subjects completed functional magnetic resonance imaging (fMRI) scans before and 1 hour after the two stress tests. Cognition was assessed in the fMRI scanner using the N-back working memory paradigm. Serial pain and cardiovascular assessments were obtained throughout the protocol. We defined two exercise induced phenotypes of GWI based upon orthostatic tachycardia and systemic hyperalgesia. Subgroup identification was associated with static anatomical differences in white and gray matter, baseline patterns of blood oxygen level dependent (BOLD) flow during cognitive testing, and significant dynamic exercise-induced changes in BOLD patterns in working memory, attention and pain processing networks.

## Results

### Phenotype Identification

**Figure 1:**
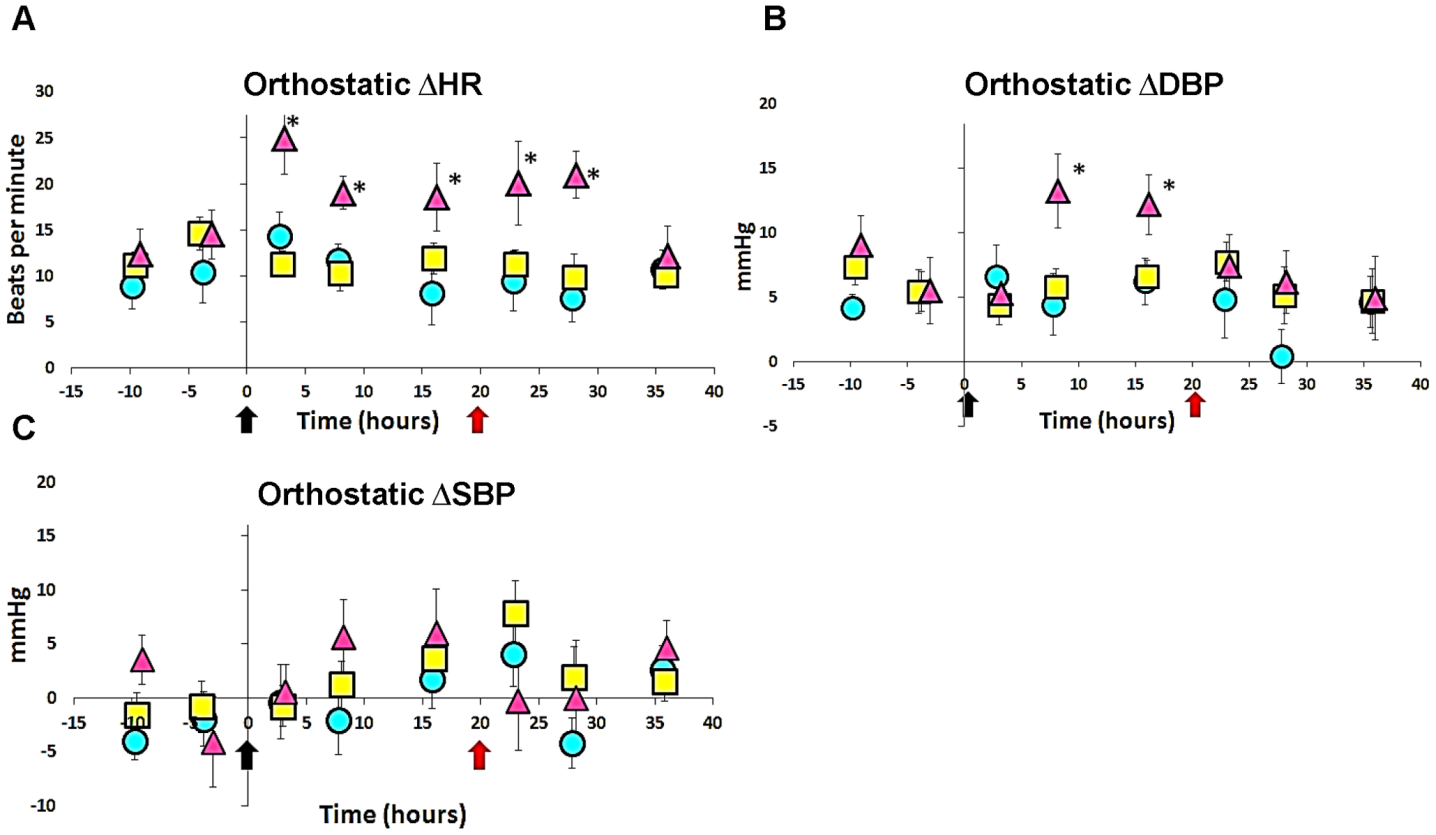
Exercise elicits orthostatic tachycardia and diastolic hypertension. (**A**) Prior to first exercise, all participants had equivalent ΔHR. Three hours after the first stress test, 10 GWI subjects met the criteria for orthostatic tachycardia (START phenotype; *P* = 0.037, Fisher's test). START (*n* = 10; magenta triangles) subjects compared to controls (*n* = 10; cyan circles) and STOPP (*n* = 18; yellow squares) continued to have higher ΔHR recordings at 8, 16, 24 and 29 hours after first exercise. (**B**) START had significant increase ΔDBP at 8 and 16 hours after the first stress test. (**C**) Exercise did not cause ΔSBP in any of the subgroups. Δ = postural change. SBP = systolic blood pressure DBP = diastolic blood pressure HR = heart rate. Black and red arrows are start of first and second stress test respectively.*P<0.001; Error bars are means ±95% C.I.

There were no significant differences in demographic variables or Gulf War – related exposures between GWI and control subjects (Table A in File S1). All GWI subjects also met criteria for CFS [14]. Day 1 was an acclimatization period that included all history, physical, and baseline studies (Table B in File S1). Supine and standing heart rate (HR), systolic (SBP) and diastolic blood pressure (DBP) and their associated postural (orthostatic) changes were equivalent between GWI and controls (Figure 1 and Table C in File S1). Postural (orthostatic) changes are defined as the difference between the supine and standing measurement in the cardiovascular indices (ΔHR, ΔSBP and ΔDBP).

On day 2 all subjects completed their pre-provocation fMRI followed by their first exercise. Three hours after the first exercise test, ten GWI subjects (*P = *0.037 vs. controls, Fishers Exact test; Table D in File S1) met the criteria for postural orthostatic tachycardia. This subgroup was identified as the Stress Test Associated Reversible Tachycardia (START) phenotype.

To assess changes in pain perception, we used digital palpation of approximately 4 kg of pressure at 18 traditional fibromyalgia tender point sites [18]. Sixty-eight percent (19 out of 28) of the GWI subjects met the criteria for fibromyalgia which is consistent with reports of increased prevalence [19]. We repeated this digital palpation to assess longitudinal changes. At baseline controls had significantly fewer positive tender points than GWI subjects (*F*_2,35_ *= *5.58, *P = *0.007; Figure A in File S1). This observation was consistent throughout the protocol.

GWI subjects (*n* = 18) with no evidence of exercise induced postural tachycardia, had a significant increase in positive tender points after the two exercises compared to baseline (*P = *0.007, 2-tailed paired t-test; Figure A in File S1). This subgroup was termed the Stress Test Occurring Phantom Perception (STOPP) phenotype. START subjects did not have a significant increase in positive tender points over the 4 day period. The remainder of the analysis is discussed for the phenotypic START, STOPP and control subgroups.

### Changes in Cardiovascular Indices

START subjects average ΔHR 3 hours following the 1^st^ exercise was 24.9 [21.0 to 28.8]; (mean [±95% CI]) beats per minute compared to controls (14.3 [11.7 to 16.9]) and STOPP (11.3 [12.9 to 15.7]). The significant ΔHR persisted after the 2^nd^ exercise test and finally resolved by the 4^th^ day (Figure 1A).

Noteworthy changes in postural heart rate (ΔHR) were tested using type 3 analysis of deviance with the factors GROUP (controls vs. START vs. STOPP) and TREATMENT (pre-exercise vs. post exercise). This revealed an effect for GROUP *x* TREATMENT (*x^2^* (2) = 8.81, *P<*0.037). Specifically, all pairwise comparisons with Bonferroni corrections indicated significant increases post-exercise between START and controls (Z = 4.13, *P<*0.0006) and START and STOPP (Z = 4.15, *P<*0.0005) but not STOPP and controls. No significant ΔHR differences between groups were found pre-exercise. START subjects also had a significant increase in postural diastolic blood pressure (ΔDBP) 8 hours after the 1^st^ exercise (*F*_2,166_ = 17.3, *P = *0.000004; Figure 1B) with an average ΔDBP of 13.4 [10.5 to 16.3] mmHg. START had no significant increases in postural systolic blood pressure (ΔSBP) (Figure 1C). Control and STOPP groups did not exhibit any postural tachycardia, systolic or diastolic hypertensive changes after exercise (Figure 1A, 1B, and 1C). Exercise did not cause changes in supine (lying down) HR, SBP, or DBP in any subgroups (Table C in File S1). .

### Self-reported Questionnaires

At baseline, START subjects self-reported higher interoceptive complaints (*F*_2,34_ = 16.8, *P = *0.000008) and anxiety (*F*_2,34_ = 16.04, *P = *0.000012) than STOPP and controls providing a correlate to the phenotype (Table E and F in File S1). There were no significant differences in fatigue scores between START and STOPP subgroups but both had significantly greater fatigue ratings than controls (Table G in File S1). START and STOPP subjects had lower SF-36 domain scores than controls (Table H in File S1).

In a broader validation cohort, principal component analysis (PCA) with Monte Carlo simulation of the Chalder’s fatigue questionnaire [20] indicated START subjects loaded a primary mental (cognitive) construct whereas STOPP subjects loaded a primary physical construct (Table I in File S1). Such distinct loadings between START and STOPP subgroups suggest different pathophysiological mechanisms may underlie the perception of fatigue.

### Working Memory Function before Exercises

**Figure 2:**
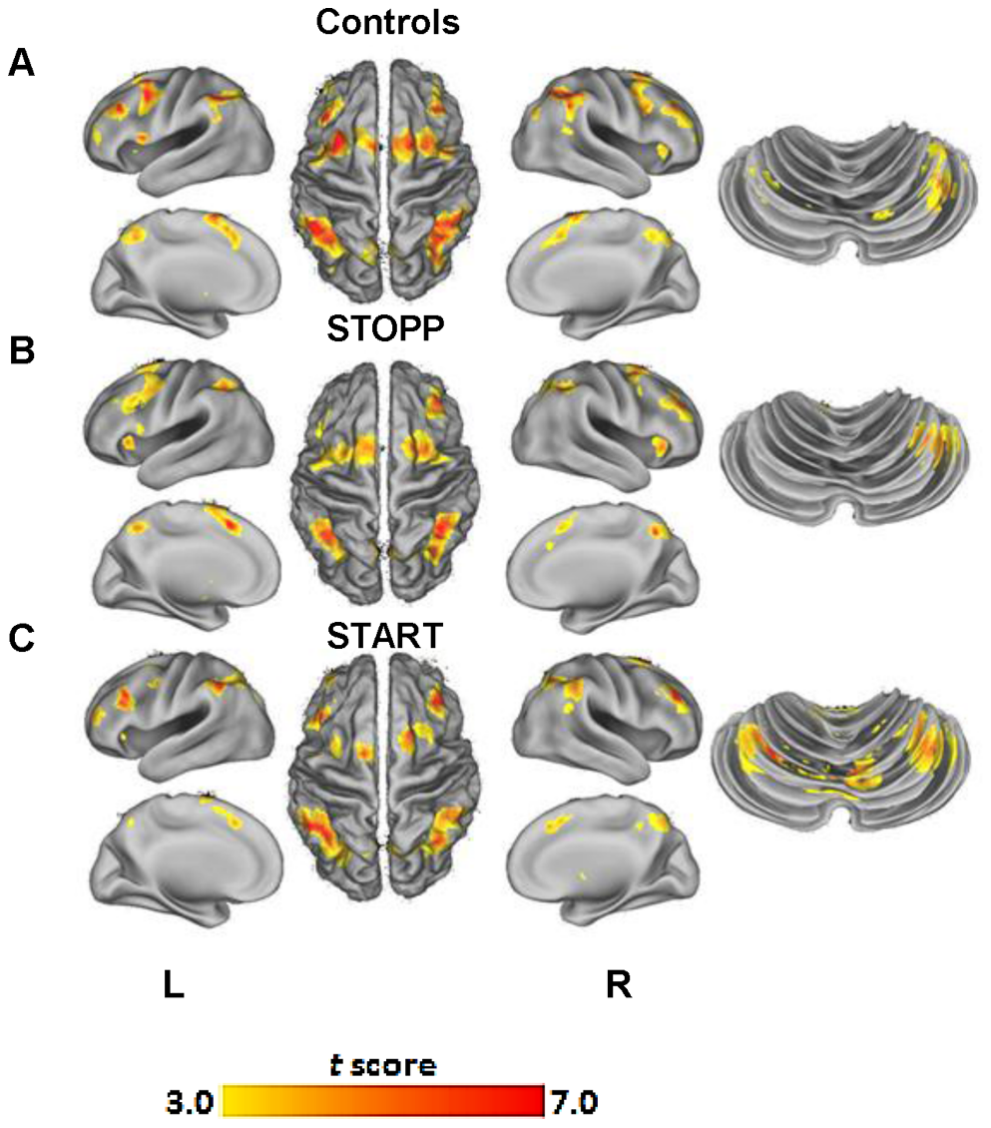
Pre-exercise compensatory activation during cognitive task. (**A**) Controls significantly activated regions normally associated with working memory in the frontal and parietal lobes and basal ganglia. (**B**) STOPP subjects activated normal working memory regions in addition to significant areas in bilateral anterior insula and right caudate body. (**C**) START subjects activated cortical regions associated with working memory and extensive activation of bilateral posterior-lateral cerebellum and right vermis. Whole-brain maps are displayed at *P<*0.001 and were cluster corrected for multiple comparisons at *P<*0.05 using AlphaSim.

The letter variant of the 2-back task was used to gauge verbal working memory [21]–[25]. Within group analysis of baseline BOLD activity revealed GWI subgroups and control subjects activated regions consistent with the frontal-parietal network (FPN) that subserves working memory and attention (*P*<0.05; false discovery rate (FDR); Figure 2) [21], [26]–[28]. Controls and STOPP subjects activated striatal regions. In contrast, START subjects did not have striatal activity but prominent bilateral activation in the cerebellar vermis (Table J in File S1). This is consistent with functional compensation seen in other states of neurodegeneration [29]–[31]. Direct between group comparisons showed controls activated the left middle frontal gyrus and left precuneus (*P*<0.05, AlphaSim; Table K in File S1). STOPP subjects recruited the left superior frontal gyrus, bilateral anterior insula, right precuneus, left caudate body, and right cerebellar tuber. In contrast, START subjects activated the right medial frontal gyrus, right superior parietal lobule, left cerebellar pyramis, and right cerebellar culmen. BOLD activity of compensatory working memory, obtained before exercise, correlated distinctly with the phenotypic designation of START and STOPP. Both GWI subgroups had significantly lower accuracies compared to controls (*F*_2,35_ = 5.12, *P = *0.011; Figure B in File S1).

### Working Memory Function after Exercises

**Figure 3:**
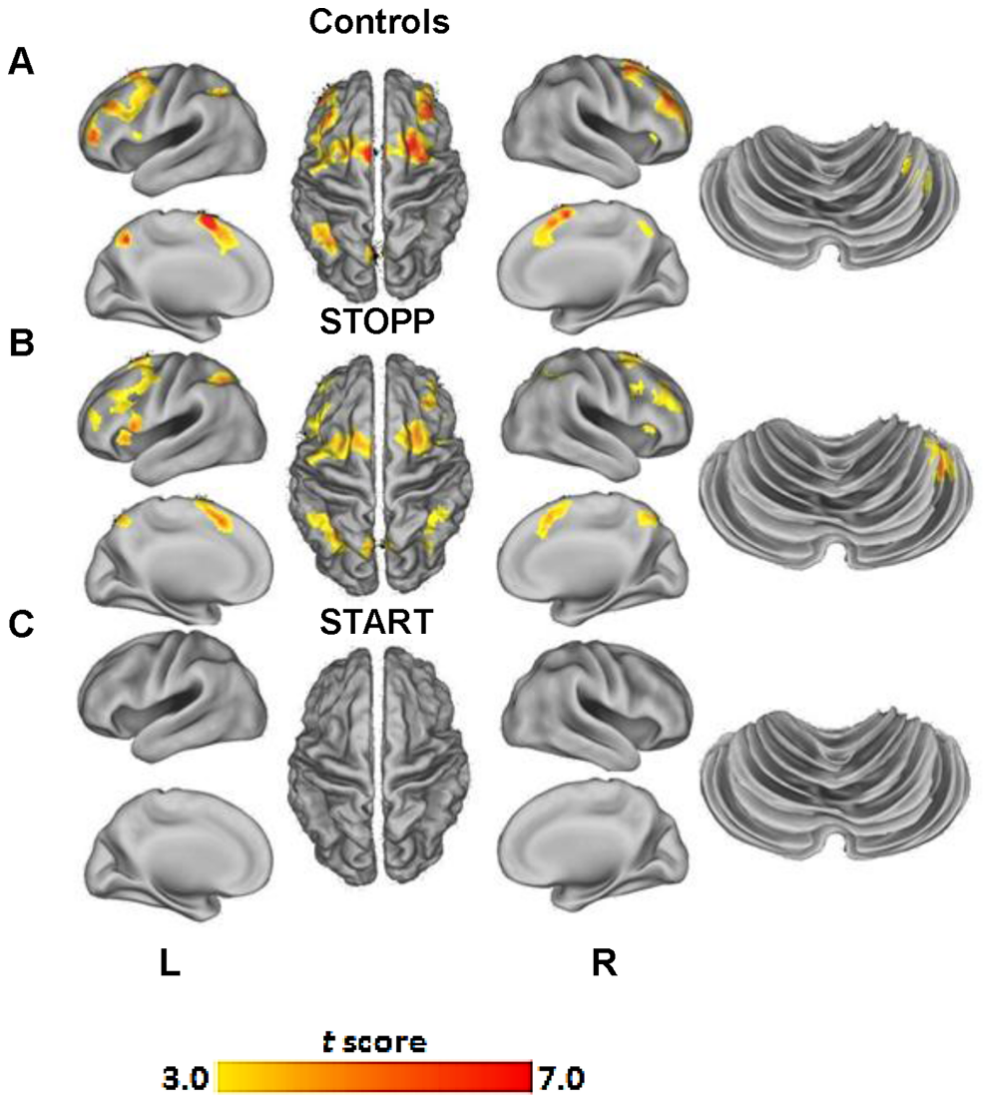
Acute exercise prompts changes in neural networks observed during cognitive task. Exercise reduced cerebral and cerebellar activity for all groups. (**A**) Controls activated areas in agreement with normal working memory function in the frontal and parietal lobes. (**B**) STOPP subjects activated normal working memory regions as well as compensatory right cerebellar recruitment. (**C**) The largest decrement in activity was in the START group that had no net change in regional blood flow to the cerebrum or cerebellum. Whole-brain maps are displayed at *P<*0.001 and were cluster corrected for multiple comparisons at *P<*0.05 using AlphaSim.

Subjects completed a post-exertional fMRI scan within 1 hour of the second exercise test. Within group analysis revealed that controls had BOLD activity consistent with the FPN implying exercise did not affect working memory capabilities (*P*<0.05, FDR; Figure 3A). STOPP subjects recruited similar FPN areas but with additional activation within the left medial frontal gyrus, bilateral anterior insula and cerebellum (Figure 3B). In stark contrast, START subjects did not significantly activate any regions (Figure 3C). Further exploratory analysis (*P*<0.01, whole-brain uncorrected) did not reveal any other BOLD activity (Table L in File S1).

Direct between group comparisons showed controls activated their bilateral superior frontal gyrus, left middle frontal gyrus, left precuneus and left inferior parietal lobule (*P*<0.05; AlphaSim). STOPP subjects activated left medial frontal gyrus, bilateral superior parietal lobule, bilateral middle frontal gyrus, and right cerebellar tonsil. Exercise significantly altered functional BOLD activity in the START and STOPP subgroups (Table M in File S1). 2-back accuracies were still significantly lower than controls (*F*_2,35_ = 5.22, *P = *0.018; Figure B in File S1).

### Alterations in White Matter Tracts

Working memory tasks require communication between the prefrontal and parietal lobes [21]. These regions communicate via the bilateral superior longitudinal fasciculus (SLF) [23], [24], [32]. We hypothesized that the variations in N-back scores may be related to alterations in the bilateral SLF. Using diffusion tensor imaging (DTI), each individual's fractional anisotropy (FA) was calculated. FA is a general index for the integrity of white matter and is a direct marker for injury [33], [34].

**Figure 4:**
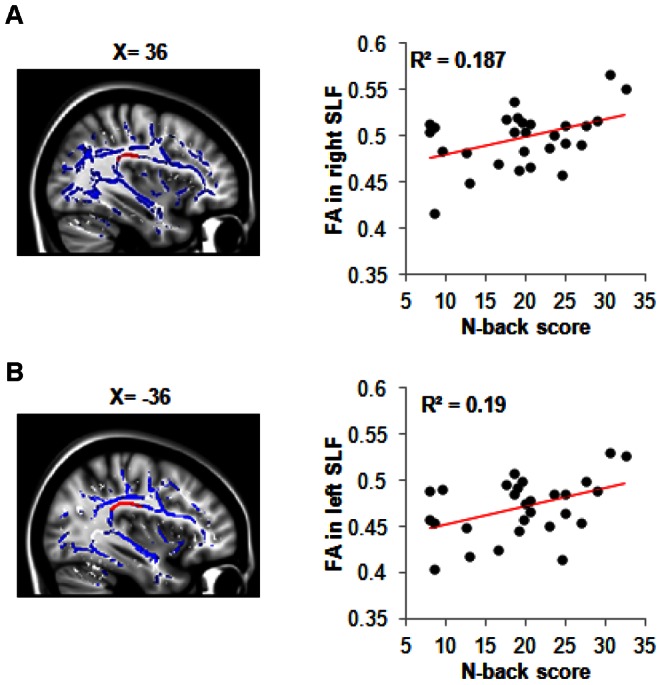
Relationship of white matter FA with working memory scores. (**A**) Sagittal view of the right superior longitudinal fasciculus (SLF) ROI’s (red) overlaid onto the mean FA tract skeleton for GWI subjects (*n* = 28), with scatterplot showing a relationship between right SLF and mean 2-back score (*P* = 0.010) (**B**) Sagittal view of left SLF ROI (red) overlaid onto the mean FA tract skeleton for GWI subjects (*n* = 28), with scatterplot showing a relationship between left SLF and mean 2-back score (*P* = 0.011).

FA positively correlated with 2-back scores across all participants in the right (*r = *0.365, *P = *0.012) and left (*r = *0.347, *P = *0.016) SLF. Further analysis excluding controls identified stronger correlations in the right (*r = *0.432, *P = *0.010; Figure 4A) and left (*r = *0.436, *P = *0.011; Figure 4B) SLF. There were no differences in FA values for the bilateral SLF between the groups (Table N in File S1). This suggests that cognitive dysfunction in GWI may be related to individual variations in bilateral SLF white matter integrity.

### Brain Volume Differences

**Figure 5:**
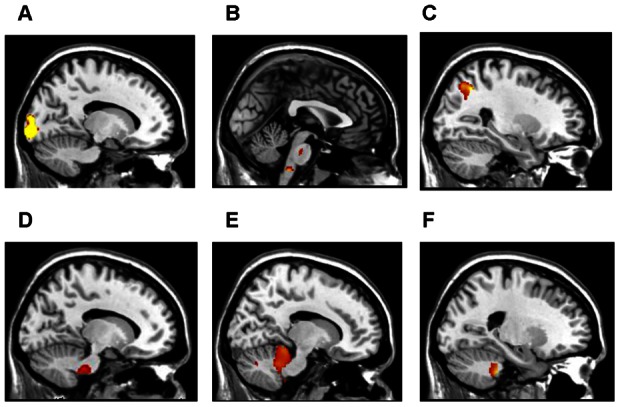
Phenotype designation reveals differences in gray matter and white matter density. (**A**) START subjects (in contrast to controls) had less gray matter volume in the left lingual gyrus extending into the left cuneus (*P*<0.025) and (**B**) right pons and right medulla (*P*<0.02) (**C**) STOPP subjects (in contrast to controls) demonstrated a trend of less gray matter in the right superior parietal lobule extending into the right precuneus (*P*<0.07). (**D**) START subjects had reduced white matter volume (in contrast to STOPP) in the left pons (*P*<0.004) and (**E)** left cerebellar tonsil and left pyramis (*P*<0.012) (**F**) Analyses also demonstrated START subjects (in contrast to STOPP) had decreased gray matter in the right culmen extending into the right fastigial and left dentate nucleus of the cerebellum (*P*<0.035). All *P* values are corrected for age, gender and multiple comparisons using non-stationary cluster correction.

Regional volume differences were identified using voxel based morphometry (VBM) with gender and age as confounds (Table O in File S1). START subjects had less neocortical gray matter volumes compared to controls in the left lingual gyrus (*t*_16_ = 5.42, *P*<0.025; Figure 5A), right pons and right medulla (*t*_16_ = 3.97, *P*<0.02; Figure 5B). STOPP subjects had less robust differences with decreased cortical gray matter volume than controls in the right superior parietal lobule (*t*_19_ = 4.45, *P*<0.07; Figure 5C). Comparing GWI subgroups, START subjects had a decrease in white matter volume compared to STOPP in the left pons (*t*_25_ = 3.69, *P*<0.004; Figure 5D) and left cerebellar tonsil and left pyramis (*t*_25_ = 3.88, *P*<0.012; Figure 5E). START had a smaller gray matter volume for the right cerebellar culmen than STOPP subjects (*t*_25_ = 3.51, *P*<0.035; Figure 5F). These alterations in brainstem, cerebellar and cortical brain morphology may contribute to the unique clinical characteristics associated with START and STOPP phenotypes.

## Discussion

We demonstrate that clinical markers of pain and autonomic dysregulation are associated with dysfunction of cognitive processing and altered brain morphology in GWI. This strongly implicates dysfunction in the central nervous system is related to chronic symptom complaints in Gulf War Illness. Our results support the use of exercise as a potential diagnostic tool to verify case designation criteria and divide GWI into two phenotypic variants. Linking clinical markers to alterations in the brain extends our understanding of the underlying mechanisms in GWI.

The original hypothesis proposed that stress tests would alter symptoms in Gulf War veterans but not controls and allow for the identification of underlying central mechanisms and regions of dysfunction. We did not anticipate discrete changes indicating two GWI subgroups. These findings prompted further analyses into exercise-induced symptoms and its relationship to structural and functional brain parameters.

### Clinical Findings

Measurements of orthostatic intolerance and auto-regulatory dysfunction have been inconsistent in GWI [35], [36]. Differences in findings may be due to small intra-subject recording samples and/or different procedures used, such as tilt table testing [35]. To overcome this obstacle, we studied autonomic function throughout the four day period. Prior to the first stress test, all participants had equivalent cardiovascular measures.

Exercise stress tests were sufficient to cause START subjects to have orthostatic tachycardia; defined as an increase in heart rate from the supine to upright position of more than 30 beats per minute on two measurements [37]. START subjects were not diagnosed with postural orthostatic tachycardia syndrome (POTS) because none had a previous diagnosis, any observed pre-exercise symptoms, and chronotropic alterations were reversible because they abated on the 4^th^ day.

Standing up from a recumbent posture modestly elevates HR and DBP [38]. Curiously START subjects did not have any significant increase in ΔDBP 3 hours after the 1^st^ exercise at the time of the observed tachycardia. Instead ΔDBP developed 8 hours after the first exercise. The mechanism leading to the disassociation between ΔHR and ΔDBP regulation was not apparent. We suggest that fatiguing exercise affected the START participants’ baroreceptor reflex arc leading to postural tachycardia and the delayed onset of diastolic hypertensive effects.

At baseline, both START and STOPP had significantly more positive tender points than controls. Exercise caused the STOPP group to have significantly more tender points. In contrast, acute exercise in healthy subjects is known to cause a temporary decrease in pain perception leading to exercise induced hypoalgesia [39]. Exercised induced hyperalgesia has been found in GWI and CFS which is consistent with our findings [17], [40]. Separating subjects into groups based upon a stressor response may generate more homogenous subgroups for study of mechanisms and treatments.

Consistent with previous reports, GWI subjects had significantly higher self-reported symptoms of fatigue, lower quality of life and other functional complaints than controls at baseline [2]–[5], [19]. The START and STOPP phenotype had significantly higher interoceptive scores than controls. However, START subjects had higher anxiety scores than STOPP and control subjects. This implies START subjects uniquely have increased awareness of interoceptive bodily processes that are often co-morbid with anxiety [41], [42]. This outcome may have clinical relevance. For example, low doses of GABAergic anxiolytics may be beneficial in the setting of psycho therapeutic support with appropriate medical monitoring. The absence of significant anxiety in STOPP subjects is equally important, since phenotypic identification may shield subjects from side effects related to unnecessary medications. Extensive future validation is warranted.

Fatigue is a common complaint in GWI and has been defined as a subjective lack of physical and/or mental energy perceived to interfere with normal activities [4], [5], [14]. This dichotomy was revealed by PCA analysis of the Chalder’s questionnaire where a primary cognitive construct was identified for START and a physical construct for STOPP. Others have only found a primary physical fatigue construct [20], [43]. This is the first study to discriminate subgroups in GWI based on the primary type of fatigue and suggests it may have different driving components.

Like other studies our GWI subjects had worse cognitive function than controls [44], [45]. However, this was not replicated in a large population based sample [46]. Although, our sample size is small and has subgroups, our study cohorts are representative of the 1995 Gulf War National Health Survey (Table A in File S1) and other population based studies [1], [19], [46].

### BOLD Activity before Exercises

STOPP subjects showed compensatory BOLD activity in the striatum before exercise. The striatum is implicated in attentional shifting, focus, and learning [47], [48]. STOPP subjects had increased activity within the right precuneus which is consistent with their perceived fatigue [49]. Activation of the bilateral anterior insula corresponds to integrated interoceptive and nociceptive signals from pain, and/or distress [50]–[53]. These perceptions may distract from efficient working memory [54]. In fact, greater utilization of the left striatal caudate by STOPP may reflect increased compensatory effort to resist such distracters [47].

In contrast, START subjects did not activate any striatal areas, prior to exercise, but exhibited bilateral BOLD activation in the cerebellar vermis indicating functional compensation as seen in states of neurodegeneration [29]–[31]. The cerebellar vermis is involved in phonological rehearsal, a key component of verbal working memory [55]. However, even with compensatory neural activity, GWI subjects had lower working memory accuracy than controls. This is consistent with the theory that compensation is limited by structural damage [56] and is highlighted by the correlation observed between accuracy and white matter FA values in the bilateral SLF.

### BOLD Activity after Exercises

After exercise, controls maintained normal working memory and frontoparietal function [57]. STOPP shifted their major regions of activation from the superior frontal gyrus to the left medial frontal gyrus. START subjects did not significantly recruit any regions, during the cognitive task. Fatiguing activity is known to decrease blood flow to cerebellar and cortical regions [58]. It is plausible that exercise disrupted the cerebellar compensatory function leading to loss of BOLD activity. In addition, exercise may have affected autonomic control systems leading to orthostatic tachycardia.

### Structural Brain Changes

It is often difficult to confirm discrete pathophysiology for subgroups if significant differences may extend across a spectrum. However, our patterns of gray and white matter loss were unique to phenotypes. STOPP subjects had a trend for decrease gray matter volume in the right superior parietal lobule. This region is adjacent to activation areas known to process pain and may have led to the increase in positive tender points following exercise [59]. Gray matter atrophy is consistently associated with chronic pain syndromes [60].

START subjects exhibited volume loss in the left lingual gyrus. This region promotes efficient cognition [61], so that atrophy may have contributed to lower 2-back scores. Cerebellar and brainstem atrophy may have been vulnerable to the exercise challenges, leading to orthostatic tachycardia and exercise induced loss of working memory BOLD activity [62]–[68].

Our study has limitations. As with any statistical analysis using correlation, we cannot establish whether the relationship between white matter and cognitive abilities reflects a causal correlation. This is also true for fMRI data. Observed differences in working memory scores compared to other studies could be attributed to the use of a different paradigm. Lack of functional activity after exercise in START subjects may be due to the masking procedure used during fMRI analysis. Future studies should examine whole brain blood flow to address this possibility. Further replication and validation is needed.

In conclusion, static and dynamic exercise-induced neurocognitive changes were observed that defined 2 GWI phenotypes. Investigation of mechanisms (Table P in File S1) may help explain current criteria [1]–[6]. Exertional exhaustion, elicited by our testing paradigm, has been a hallmark complaint of other idiopathic illnesses [12]–[16]. Therefore our findings are likely to have broad relevance.

Potential changes in procedures related to clinical diagnosis should try to incorporate active stressors to uncover compensated physiological deficits with confirmation from testing methods such as postural tachycardia, hyperalgesia, and fMRI to assess brain structure and function. Identifying biomarkers such as these for phenotypic designation is one way to begin untangling the pathophysiological and molecular mechanisms underlying idiopathic disease states such as Gulf War Illness.

## Materials and Methods

### Ethics Statement

This protocol was approved by the Georgetown University Institutional Review Board (IRB 2009-229) and USAMRMC Human Research Protection Office (HRPO #A-15547.0), and listed in clinicaltrials.gov (ID#: NCT01291758). Healthy and Gulf War Illness (GWI) veterans from the 1st Gulf War, and healthy, non-military control subjects were recruited between 2009 and 2011 from websites, word of mouth, fliers, newspaper and on-line advertisements, and personal contacts in clinics and support groups. All subjects signed informed consent.

### Subjects

A total of 250 interested participants responded via telephone or electronic mail (http://www9.georgetown.edu/faculty/baraniuj/Site/2009-229.html). Each volunteer had an initial telephone screening with a clinical research associate who read a scripted outline of the study and answered specific questions. After giving verbal consent, candidates were screened for military service for at least 30 consecutive days between August 1, 1990 and July 31, 1991; service in the Gulf War Theatre; Veterans Administration diagnosis of Gulf War Illness and other related ratings and disabilities; CDC criteria for GWI [4] and Chronic Fatigue Syndrome (CFS) [14]; current medications; chronic medical and psychiatric illnesses; and factors preventing functional magnetic resonance imaging (fMRI).

Exclusion criteria included active duty military personnel; current medical, neoplastic, or psychiatric conditions that could be associated with fatigue and other GWI and CFS complaints; HIV or viral hepatitis infection; pregnancy or lactation; previous myocardial infarction, arrhythmia, amputation or other physical limitation that would preclude exercise stress testing; use of medications that could interfere with exercise stress testing or brain blood flow and that could not be tapered or discontinued (e.g. beta-blockers, antipsychotics); claustrophobia; ferrous - based implants, prostheses or stents; metallic tattoos; known fear of needles or difficulties drawing blood.

### Pre-arrival Directions and Questionnaires

Sixty-six subjects were given approval to participate and completed pre arrival questionnaires on our online eZhengtricity data collection system (http://www9.georgetown.edu/faculty/baraniuj/Site/2009-229.html) No personal identifying information was collected using this system. Questionnaire results were collected in real-time, and downloaded to back-up and excel databases for further confidential analysis. Eleven subjects withdrew without completing questionnaires. Five completed the questionnaires then withdrew.

The Chalder Fatigue Score was used to verify presence of fatigue [20]. Quality of life (disability) was assessed by the Medical Outcomes Survey Short Form 36 (MOS-SF-36) [69]. Other questionnaires addressed the presence and severities of airway, bowel, bladder symptoms, anxiety and sensitivities to chemical exposures [70]–[78]. Once online questionnaires were completed, subjects were scheduled for the 4 day protocol and instructions were provided on logical means to taper specific medications prior to arrival at the Georgetown University Clinical Research Unit (CTSA-CRU).

### Arrival, Screening Day, and Orthostatic Measurements

During their first day, subjects had a screening visit that included their first orthostatic measurements of vital signs. The change in heart rate (ΔHR, beats per minute), diastolic (ΔDBP, mmHg) and systolic blood pressures (ΔSBP, mmHg) were calculated. Subjects rested supine for 5 min. Vital signs were measured by a calibrated, automated blood pressure cuff (Dinamap 300). Subjects then stood up with their heels 10 inches away from a wall. Vital signs were measured every minute for the next 5 minutes.

Postural (supine to standing) tachycardia was defined as an elevation of Δ≥30 beats per minute on 2 occasions [37], [79]. Postural systolic hypertension or hypotension was defined as an increase or decrease of Δ≥20 mm Hg from recumbent on 2 measurements respectively [38], [79]. Postural diastolic hypertension was defined by receiver – operator analysis for this GWI population as an increase of Δ≥18 mm Hg on 2 occasions. This threshold had a specificity of 0.83 and sensitivity of 0.80 (area under the curve = 0.85) (Figure C and Table Q in File S1). Postural measurements were performed a total of 8 time points throughout the protocol (Table B in File S1).

### Bicycle Stress Tests

Two protocols were evaluated. First, standard VO2MAX cardiopulmonary stress tests were performed using Vmax equipment and software (SensorMedix) [80], [81]. Second, subjects had modified stress tests of 25 minute duration at 70% predicted hear rate followed by a climb to 85% HR to reach anaerobic threshold (reported separately) on a Schwinn AirDyne bicycle as described for CFS subjects [16]. There were no arrhythmia, ischemia, or other alterations indicating all tests were negative as cardiac stress tests.

### Task Design

All subjects completed an N-back verbal working memory task with blocks of 0-back and 2-back loads. The task was presented as a sequence of individual uppercase letters for 1,000 ms and was separated from the upcoming stimulus by 1,500 ms of blank screen. Blocks of 9 randomized letters were presented. Subjects were given instructions to respond by pressing a button for the same letter (“0-Back”) or the one seen 2 letters previously (“2-Back”). Alternating blocks for 0-back then 2-back tasks were presented for 5 cycles. Responses were collected from a thumb-button box that was placed where the subject was able to grasp it with both hands.

Stimuli were presented using E-Prime software (Psychology Software Tools, Pittsburgh, PA) and displayed onto a screen at the head of the fMRI scanner which was seen by participants through an adjustable mirror that is attached to the head coil.

Accuracy was measured by subtracting the sum of misses and false positives from the number of condition items then dividing by the number of condition items.

Prior to entering the scanner, subjects were familiarized with the N-back paradigm through practice sessions on a standalone computer. This ensured that subjects understood the directions and expectations during the session.

### Scanning Equipment

Data was acquired on a Siemens 3 T Tim Trio scanner equipped using a transmit-receive body coil and a commercial twelve -element head coil array.

### FMRI Image Acquisition, Processing and Analysis

FMRI acquisition used T2*-weighted gradient-echo planar imaging (EPI) during the 2-back task. The blood oxygenation level dependent (BOLD) functional MRI acquisition parameters are: TR/TE 2000/30 ms, 90° flip angle, 192 mm^2^ FOV, 96×96 matrix, and 30 slices with a 3.0 mm slice thickness for an effective resolution of 3.0 mm^3^.

Image processing and statistical analysis was carried out on SPM5 software (http://www.fil.ion.ucl.ac.uk/spm/software/spm5/). SPM5 was used to correct for sequential slice timing with all images realigned to the first image to correct for head motion artifact between scans. Data was spatially smoothed using a Gaussian kernel of 5 mm full-width half maximum (FWHM). Realigned images were then mean-adjusted and spatially normalized into the Montreal Neurological Institute (MNI) standard stereotactic space.

To highlight regions of neural activity, we used a masking procedure for each portion of the paradigm; one for the 0-back and one for the 2-back. Threshold level for the masking procedure was set at *P*<0.001 uncorrected. Then, further analysis used a fixed-effects single subject rendering during the 2-back task in contrast with the 0-back task (2-back>0-back contrast). This is followed by a second-level investigation that uses a random-effects group analysis (one-sample t-tests) on the summary statistical images from the first-level analysis. All statistical tests were entered into the design matrix as covariates to control for age and gender related differences. All reported ROIs at the voxel level were corrected (*P<*0.05) using FDR. Finally, the resulting activation maps were displayed onto an anatomically standardized mean T1 image of all the subjects and then rendered onto the standard caret brain with corresponding T-value scaling [82].

To identify significant regions of BOLD activity between groups and across days, we used the AFNI based AlphaSim program, a Monte Carlo simulation (http://afni.nimh.nih.gov/pub/dist/doc/manual/AlphaSim.pdf). For a given voxel-wise probability threshold and the given search space of the whole brain, this method derives the cluster volume needed to hold the false-positive rate for cluster detection at a desired level. Using a voxel-wise threshold of *P*<0.001 uncorrected, a cluster volume threshold of 90 contiguous voxels and smoothness with a FWHM of 13 mm^3^ renders 90 voxels was significant to hold the probability of map-wise false-positive detection at *P<*0.05 in the whole-brain analyses.

### VBM Image Acquisition, Processing and Analysis

Structural 3D T1-weighted MPRAGE images parameters were: TE = 2.52 ms, TR = 1900 ms, TI = 900 ms, FOV = 250 mm, 176 slices, slice resolution = 1.0 mm, voxel size 1×1×1 mm. All MPRAGE images were processed using SPM8 (http://www.fil.ion.ucl.ac.uk/spm/software/spm8/) on MATLAB.

VBM was completed using the DARTEL toolbox for SPM [83], [84]. Images were segmented into gray matter, white matter, and cerebrospinal spinal fluid. The gray and white matter images from all subjects were then simultaneously registered together, and a study specific template was created from the average MRI space to reduce between-subject variability. The template was used to normalize images into the standard MNI template space. DARTEL “preserve amount” option was used to retain the volumetric data of the original images that were smoothed with a Gaussian kernel with 12-mm FWHM [85].

All statistical analysis for VBM was carried out using the SPM8 software package. Whole brain analyses were conducted on both gray and white matter of cortical areas only with an uncorrected p-value of *P<*0.001. ROI masks of gray and white matter were used for the brainstem and cerebellum which were created from the a priori images from SPM8 and the Talairach Daemon hemispheres option found in the WFU PickAtlas (version 3.0.3) toolbox [86], [87]. A minimum intensity threshold of 0.45 was used to help insure that a higher probability of gray or white matter was obtained.

These apriori masks were then separately applied to the GWI and control subjects thresholded gray and white matter images leading to exclusive masks for our subject populations These masks were then applied with all subsequent statistical tests. For all analyses, whole brain and ROI, a minimum voxel intensity threshold of 0.2 was also applied to the subjects’ data with the statistical tests to help insure maximum content of grey or white matter. The intracranial brain volume was calculated by combining the brain volumes found in each subject’s native space grey matter, white matter, and CSF images. Then, to account for global brain volume differences, each subject’s intracranial brain volume was used to normalize the global differences. Finally, for all statistical tests, subjects’ age and gender were entered into the design matrix as covariates to control for age and gender related differences in grey and white matter volumes.

Independent 2 sample t-tests were performed comparing the control, STOPP, and START groupings. For the whole brain analyses, an underlying uncorrected *P<*0.001 was used, and for the ROI analyses, *P<*0.005. Statistical tests were performed on the cluster level using the Non-Stationary Cluster Extent Correction toolbox for SPM with a corrected value of *P<*0.05 [88]–[91]. All MNI coordinates were converted to Talairach and Talairach Daemon applet to identify significant regions and coordinates [92], [93].

### DTI Acquisition and Analysis

Two DTI scans were obtained for each subject with parameters of TE = 101 ms, TR = 7900 ms, FOV = 240 mm, 55 slices, slice resolution = 2.5 mm, voxel size = 2.5×2.5×2.5 mm. For each scan, 5 non-diffusion weighted volumes (b = 0 s/mm2) and 30 diffusion-weighted volumes (b = 1000 s/mm2) were acquired. For each subject, the two DTI scans were concatenated together to increase the signal-to-noise ratio.

Processing of the individual subject’s DTI data was performed with the TORTOISE (version 1.1.2) processing pipeline [94]. Default settings were used except where noted otherwise. First, eddy current distortion and motion correction were applied [94]. Next, susceptibility-induced EPI distortion correction was performed using the first B0 image as a target for registration [95]. Then, rigid reorientation was applied to the subject’s diffusion weighted images (DWIs), bringing them into a common final space as defined by the registered first B0 image. All corrections were performed in the native space of the DWIs, transformations were applied in a single interpolation step, and the b-matrix was reoriented appropriately [96]. In preparation to calculate the fractional anisotropy (FA) image, the signal standard deviation was calculated with the automatic method option, and then the DWIs were masked with the masking tool. Lastly, the FA and eigenvalue images were calculated using the iRESTORE algorithm provided with TORTOISE, which is a non-linear least squares method of tensor estimation [97].

The subjects' FA data were imported into Tract based Spatial Statistics (TBSS), and then aligned into a common space using the nonlinear registration tool FNIRT [98]–[101], which uses a b-spline representation of the registration warp field [102]. Next, a mean FA image was created and thinned to create a mean FA skeleton, which represents the centers of all tracts common to the group. A minimum FA threshold value of 0.2 was used applied to the skeleton to exclude periphery tracts areas with high inter-subject variability. Each subject's aligned FA data was then projected onto this skeleton. The resulting data was fed into the randomise application [103], which is a permutation-based nonparametric inference program in FSL to perform voxelwise cross-subject statistics. For each statistical test, a total of 5000 permutations were performed to build the null distribution. To correct for multiple comparisons, Threshold-Free Cluster Enhancement (TFCE) was used [103]. For all statistical tests, subjects’ age and gender were entered into the design matrix as covariates in FA values.

To extract mean values for FA, the Johns Hopkins University white-matter tractography atlas was used [104]–[106].

### Statistical Analysis of Clinical Data

Statistical Analysis was carried out using SPSS for Windows (V.20). Fisher's exact test (2 by 2) was used to identify types of orthostatic intolerance using patient "count". The continuous postural diastolic orthostatic measurements, positive tender points, and self-reported questionnaires, between the three groups, were evaluated by one way ANOVA followed by post-hoc analysis via Tukey's Honest Significant Difference (HSD). Within group differences in tender points were assessed by 2-tailed paired t-tests with Bonferroni corrections for 6 comparisons. In addition, mean [±95% confidence intervals] were calculated.

A linear mixed effects model with random effects for PATIENT and PERIOD within PATIENT was fit by maximizing log likelihood using R with the following packages: nlme, car, and multcomp [107]–[109]. ANOVA comparing full model with fixed effects for GROUP, TREATMENT, and GROUP *x* TREATMENT interaction and reduced model without interaction term revealed full model as better fit (Table R in File S1).

ANOVA comparing full model with nested random effects (PATIENT and PERIOD within PATIENT) and models with PATIENT random effect only or no random effects revealed necessity of nested random terms. ANOVA comparing un-weighted full model with full model with weights for heteroscedastic variance revealed better fit for weighted model. Although non-normality of residuals in the weighted model was still evident, no box-cox transformation of the data was identified. Attempts to address skewness by removing outliers from each group did not alter conclusions. Hence, the weighted full model was used. Type 3 analysis of deviance and post-hoc all-pairwise contrasts with Bonferroni corrections were conducted on this model (Table S and T in File S1).

PCA was used on the Chalder’s fatigue questionnaire to identify unique variations in the perception of fatigue (physical or mental construct). Monte Carlo simulation (with 1000 permutations) was used for post hoc correction for significant eigenvalues [110]. For this analysis we used a broader clinical cohort with an additional 5 STOPP subjects who were excluded from fMRI and other clinical analysis due to scanner fitment (3), fMRI broken (1) and claustrophobia (1). All 5 subjects did complete the full exercise protocol and were labelled as the STOPP phenotype.

Mean FA skeleton values for the bilateral SLF were imported into excel to render Pearson's one-tailed correlation with mean working memory scores based on a strong a-priori hypothesis from previous studies [23], [24], [25]. The apriori hypothesis was decrease in working memory scores is positively correlated with FA of white matter tracts. Mean working memory scores were calculated from averaging together each subject’s pre and post exercise N-back score.

## Supporting Information

File S1 Figure A, Change in pain perception throughout the protocol. Figure B, Group averages for percentage correct on the 2-back working memory recall task before and after exercise. Figure C, Receiver Operator Curve for ΔDBP≥18 mm Hg after exercise. Table A, Subject demographics for controls and all Gulf War Illness subgroups. Table B, Timing of fMRI, dolorimetry, exercise tests, and orthostatic measurements. Table C, Absolute mean supine and standing measurements. Table D, Fisher's exact test for postural indices. Table E, Interoceptive complaints, chemical sensitivity questionnaires, and GAD-7. Table F, Interoceptive Global score. Table G, Chalder's Fatigue Score and it's Physical and Cognitive construct. Table H, MOS-SF-36 Quality of Life Domains for GWI subgroups and controls. Table I, Principal component analysis and Monte Carlo simulation to assess Chalder fatigue scores between START and STOPP subjects. Table J, Functional network for the 2-back WM paradigm before exercise. Table K, Significant regions of activations for direct multiple comparisons during the 2-back working memory paradigm before exercise. Table L, Functional network for the 2-back WM paradigm after exercise. Table M, Significant regions activated for direct multiple comparisons during the 2-back working memory paradigm after exercise. Table N, Mean Fractional Anisotropy (FA) Values for bilateral Superior Longitudinal Fasciculi. Table O, Significant grey matter and white matter volume reduction using VBM. Table P, Case definitions and potential consensus criteria. Table Q, Values for receiver operator curve (ROC). Table R, Model selection for mixed effects statistics. Table S, Type 3 analysis of deviance for change in heart rate by group and treatment. Table T, Post-hoc comparisons of heart rate data by patient group and treatment.
(DOCX)
