# Supplementary material for: Exercise Challenge in Gulf War Illness Reveals Two Subgroups with Altered Brain Structure and Function
Source: PLoS One. 2013 Jun 14;8(6):e63903. doi: 10.1371/journal.pone.0063903 (PMC3683000; doi:10.1371/journal.pone.0063903)
Supplement: File S1 — Figure A, Change in pain perception throughout the protocol. Figure B, Group averages for percentage correct on the 2-back working memory recall task before and after exercise. Figure C, Receiver Operator Curve for ΔDBP≥18 mm Hg after exercise. Table A, Subject demographics for controls and all Gulf War Illness subgroups. Table B, Timing of fMRI, dolorimetry, exercise tests, and orthostatic measurements. Table C, Absolute mean supine and standing measurements. Table D, Fisher's exact test for postural indices. Table E, Interoceptive complaints, chemical sensitivity questionnaires, and GAD-7. Table F, Interoceptive Global score. Table G, Chalder's Fatigue Score and it's Physical and Cognitive construct. Table H, MOS-SF-36 Quality of Life Domains for GWI subgroups and controls. Table I, Principal component analysis and Monte Carlo simulation to assess Chalder fatigue scores between START and STOPP subjects. Table J, Functional network for the 2-back WM paradigm before exercise. Table K, Significant regions of activations for direct multiple comparisons during the 2-back working memory paradigm before exercise. Table L, Functional network for the 2-back WM paradigm after exercise. Table M, Significant regions activated for direct multiple comparisons during the 2-back working memory paradigm after exercise. Table N, Mean Fractional Anisotropy (FA) Values for bilateral Superior Longitudinal Fasciculi. Table O, Significant grey matter and white matter volume reduction using VBM. Table P, Case definitions and potential consensus criteria. Table Q, Values for receiver operator curve (ROC). Table R, Model selection for mixed effects statistics. Table S, Type 3 analysis of deviance for change in heart rate by group and treatment. Table T, Post-hoc comparisons of heart rate data by patient group and treatment. (DOCX) [file pone.0063903.s001.docx]

Supporting Information for

Exercise challenge in Gulf War Illness reveals two subgroups with altered brain structure and function.

Rakib U. Rayhan^1*,^ Benson W. Stevens^2,^ Megna P. Raksit^3^, Joshua A Ripple^1^, Christian R. Timbol^1^, Oluwatoyin Adewuyi^1^ , John W. VanMeter^2^ , James N. Baraniuk^1^

^1^ Georgetown University Medical Center; Department of Medicine; Division of Rheumatology, Immunology and Allergy; Washington, District of Columbia; United States of America

^2^ Georgetown University Medical Center; Department of Neurology; Center for Functional and Molecular Imaging; Washington, District of Columbia; United States of America

^3^ Georgetown University; Department of Psychology, Cognitive Neurogenetics Laboratory; Washington, District of Columbia; United States of America

*To whom correspondence should be addressed: rur@georgetown.edu

Supporting Information Contains:

1. Figures A to C

2. Tables A-T

**1.Supplementary Figures**

*****

**++**

*****

*****

*****

**Figure A. Change in pain perception throughout the protocol**. Controls (*n=*10; cyan circles) had consistently less positive tender points throughout the protocol compared to STOPP (*n=*18; yellow squares) and START (*n=*10; magenta triangles) subgroups. Only the STOPP subgroup had a significant increase in tender points by the 4th day. (**P*< 0.01, One-way ANOVA followed by Tukey's HSD test; *^++^P*= 0.007, two-tailed paired *t* test with Bonferroni correction). Error bars are Mean [±95% C.I.].

.

**B**

**A**

******

*****

**Figure B. Group averages for percentage correct on the 2-back working memory recall task before and after exercise** (**A)** Prior to exercise controls averaged 81.4% [69 to 93.8] correct letters. STOPP and START subjects averaged 57.7% [48.6 to 66.7] and 53.7% [37.4 to 69.9] correct letters respectively. There were group differences between controls and GWI subgroups. (**B)** After exercise controls averaged 82.3% [71.0 to 94.2] correct letters; compared to the GWI subgroups. STOPP and START subjects averaged 53.7% [37.4 to 69.9] and 57.7% [48.6 to 66.7] correct letters respectively. There were group differences between controls and GWI subgroups before and after exercise (**P*= 0.011, ***P*=0.018; One-way ANOVA followed by Tukey's HSD test). Error bars are Mean [±95% C.I.].


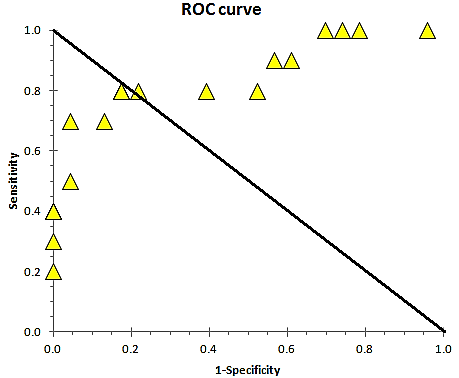


**Figure C. Receiver Operator Curve for ΔDBP ≥ 18 mm Hg after exercise.**

Receiver operator analysis of post exercise ΔDBP set the threshold between START and STOPP at Δ≥18 mmHg. This threshold had specificity of 0.826 and sensitivity of 0.80 (area under the curve = 0.85) in this unique population. The threshold was applied to the control subjects as well. The validity of the test for diastolic hypertension was based on the definition of: more than one recorded positive diastolic Δ ≥18 mmHg after standing so as to rule out blood pressure cuff error.

**2. Supplementary Tables:**

|  | C | % or M | STOPP | % or M | STOPP* | % or M | START | % or M |
| --- | --- | --- | --- | --- | --- | --- | --- | --- |
| *n=* | 10 |  | 18 |  | 23 |  | 10 |  |
| Age |  | 48.9 [± 6.1] |  | 45.8 [± 3.5] |  | 46.8 [± 2.7] |  | 44.4 [± 5.2] |
| BMI |  | 29.5 [± 3.7] |  | 31.5 [± 3.6] |  | 31.7 [± 2.8] |  | 28.5 [± 3.7] |
| Sex |  |  |  |  |  |  |  |  |
| Male | 8 | 80% | 13 | 72% | 18 | 78% | 9 | 90% |
| Female | 2 | 20% | 5 | 27% | 5 | 22% | 1 | 10% |
| Race |  |  |  |  |  |  |  |  |
| White | 9 | 90% | 14 | 77% | 18 | 78% | 8 | 80% |
| Black | 0 | 0% | 4 | 22% | 5 | 22% | 1 | 10% |
| Other | 1 | 10% | 0 | 0% | 0 | 0% | 1 | 10% |
| Branch |  |  |  |  |  |  |  |  |
| Air Force | 4 | 40% | 4 | 22% | 4 | 17% | 3 | 30% |
| Army | 1 | 10% | 10 | 55% | 14 | 61% | 5 | 50% |
| Marines | 1 | 10% | 2 | 11% | 2 | 9% | 1 | 10% |
| Navy | 0 | 0% | 3 | 16% | 3 | 13% | 1 | 10% |
| C. Guard | 0 | 0% | 0 | 0 | 0 | 0 | 0 | 0% |
| Civilians | 4 | 40% | 0 | 0 | 0 | 0 | 0 | 0% |
| **Rank |  |  |  |  |  |  |  |  |
| Enlisted | 4 | 40% | 14 | 77% | 19 | 83% | 9 | 90% |
| Officers | 2 | 20% | 4 | 22% | 4 | 17% | 1 | 10% |
| Exposures |  |  |  |  |  |  |  |  |
| Theater | 6 | 60% | 15 | 83% | 21 | 91% | 6 | 60% |
| Combat | 3 | 30% | 11 | 61% | 13 | 57% | 4 | 40% |
| Alarms | 4 | 40% | 12 | 67% | 16 | 69% | 5 | 50% |
| Oil Fires | 2 | 20% | 11 | 61% | 17 | 74% | 4 | 40% |
| ***Diesel | 3 | 30% | 13 | 72% | 16 | 70% | 8 | 80% |
| Tinnitus | 5 | 50% | 11 | 61% | 12 | 52% | 6 | 60% |

**Table A. Subject demographics for controls and all Gulf War Illness subgroups.** There were no significant differences between groups. Some Percentages do not total 100 due to rounding. Exposures are a percentage of the total population in the group. Broader validation cohort with of 5 STOPP subjects to assess robustness of START interoceptive questionnaires and fatigue scores. **Rank at discharge. *** Direct contact with >10% of skin surface and inhalation of liquid diesel, jet fuels, hydraulic fluids, paints or other volatile organic compounds for more than 30 days. Mean [± 95% C.I.].

| Study Day | Hours from 1^st^ Exercise | Activity |
| --- | --- | --- |
| Screening Day 1 | -10 | OM, dolorimetry and N-back Practice |
| **Sleep** |  |  |
| Day 2 | -3 | OM and dolorimetry |
|  | -2 | N-back practice then 1st fMRI |
|  | 0 | 1st Exercise |
|  | 3 | OM |
|  | 8 | OM |
| **Sleep** |  |  |
| Day 3 | 16 | OM and dolorimetry |
|  | 21 | 2nd Exercise |
|  | 24 | OM |
|  | 25 | N-back practice then 2nd fMRI |
|  | 29 | OM |
| **Sleep** |  |  |
| Day 4 | 36 | OM and dolorimetry |
| Discharge |  |  |

**Table B. Timing of fMRI, dolorimetry, exercise tests, and orthostatic measurements**.

During their Screening day subjects had one OM (orthostatic measurement) and 2-back practice. Every morning over the 4 day protocol, thumb pressure of approximately 4 kilograms were rendered on 18 tender points on each subject. During an OM, Subjects rested 5 minutes while recumbent, then had vital signs taken by calibrated, automated blood pressure cuffs (Dinamap 300). Subjects then stood up with their heels 10 inches away from a wall. Vital signs (heart rate and blood pressure) were measured every minute for 5 minutes. Postural changes in vital signs were calculated for each minute. On the morning of day 2 subjects had another OM followed by a 2-back practice and their first fMRI. After the completion of the fMRI, subjects prepared for their 1st bicycle exercise stress test. Three more OM’s were recorded prior to their 2nd exercise. Once the final exercise was completed patients were escorted to the Center for Functional and Molecular Imaging (CFMI) at Georgetown University Medical Center for their post-exertional 2-back practice and 2nd fMRI. Subjects had their final two OM’s after the 2nd fMRI.

|  | **Controls (*n=*10)** | **STOPP (*n=*18)** | **START (*n=*10)** |
| --- | --- | --- | --- |
| **Pre-Exercise** |  |  |  |
| Supine SBP | 127.2 [120.8 to 133.6] | 126.4 [121.6 to 128.8] | 128.4 [122.2 to 134.6] |
| Supine DBP | 73.2 [68.0 to 78.4] | 71.7 [69.5 to 75.6] | 73.6 [68.1 to 79.1] |
| Supine HR | 69.1 [66.0 to 72.2] | 68.8 [69.5 to 75.6] | 71.3 [67.4 to 75.2] |
| Standing SBP | 122.6 [119.4 to 125.8] | 123.0 [121.5 to 124.5] | 128.0 [124.7 to 131.3] |
| Standing DBP | 77.1 [74.9 to 79.3] | 77.3 [75.4 to 79.2] | 80.7 [78.1 to 83.3] |
| Standing HR | 79.8 [77.2 to 83.4] | 80.5 [77.9 to 83.1] | 85.3 [83.0 to 87.6] |
| **Post- Exercise** |  |  |  |
| Supine SBP | 125.1 [122.0 to 128.2] | 122.7 [120.9 to 124.5] | 125.1 [120.9 to 129.3] |
| Supine DBP | 71.8 [69.2 to 74.4] | 70.7 [68.9 to 73.5] | 70.5 [67.3 to 73.7] |
| Supine HR | 72.8 [70.2 to 75.4] | 72.4 [70.1 to 74.4] | 70.1 [67.5 to 72.7] |
| Standing SBP | 125.9 [124.1 to 127.7] | 121.1 [119.7 to 123.9] | 125.0 [122.3 to 127.7] |
| Standing DBP | 76.6 [75.3 to 77.9] | 75.4 [74.0 to 76.2] | 77.6 [75.7 to 79.5] |
| Standing HR | 83.6 [81.9 to 85.3] | 84.6 [82.7 to 85.3] | 91.6 [89.8 to 93.4]** |

**Table C. Absolute mean supine and standing measurements.**

During the protocol periodic measurements of postural changes were assessed. Prior to exercise there was no difference between controls and the GWI subgroups. Post hoc analysis, after phenotype subgrouping, revealed a similar situation in which there was no difference in any postural measurements. All post exercise indices were not significant except for absolute standing heart rate in the START group. (***P*<0.000001 vs. Controls and STOPP; One way ANOVA followed by post hoc Tukey’s HSD). Mean [±95% C.I].

| Group | **Pre-Exercise Orthostatic Change** | |  | **Post-Exercise Orthostatic Change** | |  |
| --- | --- | --- | --- | --- | --- | --- |
|  | Orthostatic Tachycardia | No Orthostatic Tachycardia |  | Orthostatic  Tachycardia | No Orthostatic Tachycardia |  |
| Control | 0 | 10 | *P*=0.767 | 0 | 10 | ****P*=0.037** |
| GWI | 1 | 27 |  | 10 | 18 |  |
|  | Systolic Hypertension | No systolic hypertension |  | Systolic Hypertension | No systolic hypertension |  |
| Control | 0 | 10 | *P*=1.00 | 1 | 9 | *P*=0.236 |
| GWI | 0 | 27 |  | 9 | 19 |  |
|  | Diastolic Hypertension | No diastolic hypertension |  | Diastolic Hypertension | No diastolic hypertension |  |
| Control | 0 | 10 | *P*=1.00 | 1 | 9 | *P*=0.231 |
| GWI | 0 | 27 |  | 11 | 19 |  |

**Table D. Fisher's exact test for postural indices**.

2 by 2 Fisher’s exact test was used to evaluate differences in proportions between the controls and the GWI group before and after exercise. Abnormalities at rest were no different in count or average. After exercise there was an increase in abnormal readings in which both groups had higher rates for orthostatic systolic hypertension and orthostatic diastolic hypertension but neither had a significant contingency. Rates for orthostatic tachycardia did significantly increase in the GWI group. In fact, 42 total abnormal readings (increase in postural heart rate by ∆≥30bpm) were recorded and all but one reading (41 out of 42; 98%) belonged to a group of 10 patients who had orthostatic tachycardia (subsequently named the START phenotype). All later analyses were conducted after separating out two GWI subgroups (START and STOPP) and one control group. The distribution of controls and GWI patients based upon changes before and after exercise. Systolic hypertension is defined as more than one standing reading of ∆≥20mmHg. Diastolic hypertension is defined as more than one standing reading of ∆≥18mmHg.

| **Questionnaire** | **Controls (*n*=10)** | **STOPP(*n*=18)** | **START(*n*=10)** |
| --- | --- | --- | --- |
| Rhinitis Score (0-40) | 8.5 [4.4 - 12.6] | 14.1 [10.6 - 17.6] | 23.2 [16.8- 29.6] * † |
| Dyspnea score (0-20) | 1.7 [0.2 - 3.6] | 6.7 [4.3-9.1]* | 13.2 [9.1 - 17.3] * † |
| Borg Score (0-10) | 0.50 [-0.2 -1.2] | 1.28 [0.66- 1.9] | 3.05 [2.22 - 4.28] * † |
| Gastrointestinal Construct (0-32) | 2.4 [-1.1-5.7] | 15.1 [11.1-19.1]* | 22.2 [18.0-26.4] * † |
| Urinary Construct (0-20) | 1.9 [1.2-2.6] | 5.2 [3.7-6.7]* | 10.1 [8.9-11.3] * † |
| Chemical Domain (0-100) | 15.3 [0.2 - 30.4] | 34.6 [22.7 - 46.5] | 52.8 [37.9 - 67.7] * |
| Symptoms Domain (0-100) | 15.8 [0.2- 31.6] | 43.0 [31.3 - 54.7]* | 72.3 [60.2 t- 84.4] * † |
| Impact of Sensitivities (0-100) | 8.0 [-4.9 - 20.9] | 26.7 [17.8 - 35.6] | 58.0 [40.9 - 75.1] * †† |
| **GAD-7 score** | 3.5[0.82-6.2] | 7.41 [4.9-10.0] | 14.6 [12.6-16.6] * †† |

**Table E. Interoceptive complaints, chemical sensitivity questionnaires, and GAD-7.**

START subjects had significantly higher scores for interoceptive questionnaires. (**P* ≤ 0.007 vs. Controls, † *P* ≤ 0.015 and †† *P* ≤ 0.001 vs. STOPP; One way ANOVA followed by post hoc Tukey’s HSD). Mean [±95% C.I.].

| **Interoceptive Sum** | **Controls (*n*=10)** | **STOPP (*n*=18)** | **START (*n*=10)** |
| --- | --- | --- | --- |
| Global Score (0-422) | 54.1 [3.1-105.1] | 142.4* [103.1-172.3] | 252.6** † [203.9-301.1] |

**Table F. Interoceptive Global score.** All interoceptive scores from supplementary table 5 were summed from their online questionnaires. STOPP had significantly higher scores than controls. START had significantly higher scores than STOPP and controls. (* *P* ≤ 0.012 vs. Controls, ***P* ≤ 0.0000001 vs. Controls, † *P* ≤ 0.003 vs. STOPP, One way ANOVA followed by post hoc Tukey’s HSD). Mean [±95% C.I.].

| **Domain** | **Controls (*n*=10)** | **STOPP (*n*=18)** | **START (*n*=10)** |
| --- | --- | --- | --- |
| Total Fatigue Score | 12.2 [8.9-15.5] | 24.4[20.1-28.8]* | 28.1[26.5-29.7]* |
| Physical Construct | 7.9[5.3-10.5] | 16.12 [13.8-18.5]* | 17.8[16.3-19.3]* |
| Mental Construct | 4.3[3.1-5.5] | 8.4[6.0-10.6]* | 10.3 [9.2-10.4]* |

**Table G. Chalder's Fatigue Score and it's Physical and Cognitive construct**.

Fatigue was verified using Chalder's 11-item fatigue score. Both STOPP and START subjects had higher physical construct, mental construct and total scores than controls. (**P<*0.000003 vs. Controls; One way ANOVA followed by post hoc Tukey’s HSD. Mean [±95% C.I.].

| **Domain** | **Controls (*n*=10)** | **STOPP (*n*=18)** | **START (*n*=10)** |
| --- | --- | --- | --- |
| Physical Functioning | 83.5[69.7-97.3] | 45.8[35.6-56.1]* | 37.5 [19.8-55.2]* |
| Social Functioning | 71.3[51.9-90.6] | 27.9[20.1-35.1]* | 15.0 [5.5-24.5] * |
| Role Physical | 67.5[42.1-92.9] | 14.8[0.02-29.1]* | 0.0 [0.0]* |
| Role Emotional | 86.7[69.3-104.1] | 37.3[17.1-54.3]* | 3.3[-3.2-9.8] * |
| Mental Health | 72.0[64.3-79.7] | 58.1[49.8-64.4] | 39.2[25.3-53.1]* |
| Vitality | 53.5[38.4-68.4] | 13.5[7.1-20.4]* | 14.5[7.7-21.3] * |
| Bodily Pain | 65.6[50-81.2] | 28.8[20.2-37.4]* | 17.2[7.6-26.8] * |
| General Health | 68.2[53.8-82.6] | 28.7[18.1-38.7]* | 15.7[7.5-23.9]* |

**Table H. MOS-SF-36 Quality of Life Domains for GWI subgroups and controls.** START and STOPP subjects both suffer from similar systemic complaints which lead to similarly impaired quality of life. (**P<*0.001 vs. Controls; One way ANOVA followed by post hoc Tukey’s HSD). Mean [±95% C.I.].

|  | **START (*n*=10)** | | **STOPP (*n=*23)** | |
| --- | --- | --- | --- | --- |
| **PCA loading per query** | **Component 1 †** | **Component 2 ††** | **Component 1 ‡** | **Component 2 ‡‡** |
| Poor memory | **0.921 †** | 0.038 | 0.246 | **0.657 ‡‡** |
| Difficulty concentrating | **0.801 †** | -0.557 | **0.608 ‡** | 0.491 |
| Slips of the tongue | **0.767 †** | -0.195 | 0.151 | **0.843 ‡‡** |
| Finding correct word | **0.564 †** | 0.208 | 0.008 | **0.921 ‡‡** |
| Feeling sleepy or drowsy | 0.179 | 0.040 | 0.145 | 0.089 |
| Lacking energy | -0.614 | -0.266 | **0.722 ‡** | 0.057 |
| Problems starting things | -0.589 | **0.943 ††** | -0.410 | 0.505 |
| Feel week | -0.128 | **0.835 ††** | **0.866 ‡** | -0.234 |
| Less strength | 0.102 | **0.624 ††** | **0.812 ‡** | 0.044 |
| Problems with tiredness | -0.445 | **0.573 ††** | **0.583 ‡** | 0.179 |
| Resting more | -0.280 | **0.553 ††** | **0.815 ‡** | -0.078 |
|  |  |  |  |  |
| Signifiant Eigenvalues  Variance (%)  Cumulative Variance (%) | **3.779**  34.4%  34.4% | **2.761**  25.1%  59.5% | **4.116**  37.4%  37.4% | **2.159**  19.6%  57.1% |
| Monte Carlo Simulation  Means  95th Percentile | 3.269970  **3.728901 | 2.452196  **2.691814 | 2.354251  **2.662237 | 1.844100  **2.095816 |
| Component correlation | 0.137 | | 0.178 | |

**Table I.** **Principal component analysis and Monte Carlo simulation to assess Chalder fatigue scores between START and STOPP subjects.** Principal component analysis (PCA) of Chalder fatigue score showed START subjects had a primary mental construct and secondary physical construct, whereas STOPP subjects had a primary physical construct and secondary mental construct. **eigenvalues greater than the 95th percentiles derived from Monte Carlo simulations indicated significant components.

|  |  | Controls (N=10) | | | | | STOPP (N=18) | | | | | START (N=10) | | | | |
| --- | --- | --- | --- | --- | --- | --- | --- | --- | --- | --- | --- | --- | --- | --- | --- | --- |
| Hemisphere | Location | T | BA | x | y | z | T | BA | x | y | z | T | BA | x | y | z |
| Left Cerebrum | Superior frontal gyrus | 6.51 | 6 | -5 | 8 | 55 | 7.88 | 8 | -6 | 18 | 48 | 6.51 | 6 | -12 | 0 | 70 |
|  | Middle frontal gyrus | 14.16 | 6 | -32 | 12 | 58 | 5.66 | 6 | -28 | 8 | 54 | 8.12 | 46 | -45 | 30 | 25 |
|  | Inferior frontal gyrus | 5.44 | 45 | -34 | 26 | 4 |  |  |  |  |  |  |  |  |  |  |
|  | Medial frontal gyrus |  |  |  |  |  |  |  |  |  |  | 5.03 | 8 | -5 | 20 | 44 |
|  | Pre-central gyrus | 6.71 | 44 | -50 | 8 | 8 | 4.11 | 44 | -50 | 12 | 10 | 5.95 | 6 | -46 | -4 | 40 |
|  | Superior Parietal Lobule | 5.29 | 40 | -35 | -52 | 52 | 4.41 | 7 | -30 | -54 | 48 |  |  |  |  |  |
|  | Inferior Parietal Lobule | 11.02 | 40 | -48 | -48 | 48 | 6.49 | 40 | -36 | -52 | 42 | 7.67 | 40 | -48 | -50 | 46 |
|  | Supramarginal gyrus | 5.05 | 40 | -58 | -46 | 34 |  |  |  |  |  |  |  |  |  |  |
|  | Caudate Body |  |  |  |  |  | 5.46 |  | -16 | 14 | 6 |  |  |  |  |  |
|  | Globus Pallidus Medial |  |  |  |  |  | 4.19 |  | -14 | 0 | -6 |  |  |  |  |  |
|  | Globus Pallidus Lateral | 4.34 |  | -20 | -10 | 4 |  |  |  |  |  |  |  |  |  |  |
|  | Putamen | 4.78 |  | -16 | 4 | 8 |  |  |  |  |  |  |  |  |  |  |
|  | Sup temporal Gyrus | 7.42 | 13 | -46 | -46 | 22 |  |  |  |  |  |  |  |  |  |  |
|  | Anterior Insula |  |  |  |  |  | 5.60 | 13 | -32 | 20 | 2 |  |  |  |  |  |
|  | Precuneus | 5.26 | 7 | -6 | -60 | 48 | 5.61 | 7 | -4 | -50 | -50 |  |  |  |  |  |
|  | Thalamus | 4.79 |  | -16 | -10 | 4 |  |  |  |  |  |  |  |  |  |  |
| Right Cerebrum | Superior frontal gyrus | 10.79 | 6 | 20 | 8 | 70 | 6.93 | 9 | 38 | 44 | 30 | 8.89 | 6 | 24 | 16 | 62 |
|  | Middle frontal gyrus | 7.92 | 9 | 36 | 32 | 32 | 6.34 | 9 | 38 | 35 | 35 | 5.39 | 9 | 32 | 42 | 38 |
|  | Medial frontal gyrus | 5.30 | 8 | 8 | 18 | 46 |  |  |  |  |  | 5.91 | 8 | 4 | 28 | 40 |
|  | Precentral Gyrus |  |  |  |  |  | 5.30 | 6 | 38 | -2 | 42 |  |  |  |  |  |
|  | Superior Parietal Lobule |  |  |  |  |  |  |  |  |  |  | 6.94 | 7 | 40 | -60 | 50 |
|  | Inferior Parietal Lobule | 8.76 | 40 | 34 | -46 | 44 | 7.52 | 40 | 40 | -48 | 46 | 6.71 | 40 | 50 | -40 | 44 |
|  | Insula, Anterior |  |  |  |  |  | 5.68 | 12 | 32 | 24 | -2 |  |  |  |  |  |
|  | Insula, Posterior | 4.93 | 13 | 38 | -44 | 16 |  |  |  |  |  |  |  |  |  |  |
|  | Precuneus |  |  |  |  |  | 6.95 | 7 | 8 | -60 | 48 |  |  |  |  |  |
|  | Caudate Head | 4.92 |  | 14 | 26 | -4 |  |  |  |  |  |  |  |  |  |  |
|  | Caudate Body | 4.48 |  | 22 | -6 | 24 | 3.66 |  | 16 | 16 | 8 |  |  |  |  |  |
|  | Putamen | 5.38 |  | 14 | 8 | -6 | 3.93 |  | 16 | 14 | -4 |  |  |  |  |  |
|  | Hippocampus |  |  |  |  |  | 3.78 |  | 30 | -42 | 2 |  |  |  |  |  |
|  | Parahippocampal gyrus | 4.95 | 30 | -28 | 50 | 2 |  |  |  |  |  |  |  |  |  |  |
|  | Nodule | 5.74 |  | -10 | -52 | -30 |  |  |  |  |  |  |  |  |  |  |
| Left Cerebellum | Tonsil | 5.44 |  | -34 | -56 | -32 |  |  |  |  |  |  |  |  |  |  |
|  | Culmen |  |  |  |  |  | 4.80 |  | -6 | -40 | -12 | 4.80 |  | -8 | -44 | -12 |
|  | Pyramis |  |  |  |  |  |  |  |  |  |  | 9.74 |  | -20 | -62 | -30 |
|  | Declive |  |  |  |  |  |  |  |  |  |  | 4.85 |  | -2 | -72 | -20 |
|  | Tuber |  |  |  |  |  |  |  |  |  |  | 4.51 |  | -38 | -70 | -28 |
|  | Uvula |  |  |  |  |  |  |  |  |  |  | 6.90 |  | -34 | -72 | -26 |
|  | Tuber | 6.01 |  | 38 | -64 | -28 | 6.56 |  | 34 | -58 | -30 |  |  |  |  |  |
| Right Cerebellum | Tonsil |  |  |  |  |  |  |  |  |  |  | 7.58 |  | 12 | -36 | -38 |
|  | Culmen |  |  |  |  |  |  |  |  |  |  | 5.58 |  | 34 | -60 | -26 |
|  | Pyramis | 5.27 |  | 12 | -72 | -28 |  |  |  |  |  |  |  |  |  |  |
|  | Dentate |  |  |  |  |  |  |  |  |  |  | 4.37 |  | 10 | -56 | -26 |
|  | Nodule |  |  |  |  |  |  |  |  |  |  | 4.41 |  | 10 | -52 | -28 |
|  | Declive |  |  |  |  |  |  |  |  |  |  | 5.50 |  | 32 | -52 | -32 |

**Table J. Functional network for the 2-back WM paradigm before exercise.**

Activity within the (2-Back > 0-back contrast) Controls and GWI subgroups recruited regions that are closely related to normal working memory areas. These regions activate during goal directed cognition. The subcortical activation of the basal ganglia by controls and STOPP subjects are absent in START subjects. However, START subjects activate a tremendous amount of area within the cerebellum and particularly the cerebellar vermis. (T=T value; BA=Brodmann's area; Talairach coordinates x, y and z in mm). Whole-brain maps (displayed at *P*< 0.001) were corrected for multiple comparisons at the voxel level at *P*<0.05, false discovery rate (FDR).

|  | Controls (N=10) | | | | STOPP (N=18) | | | | | | START (N=10) | | | | | | |
| --- | --- | --- | --- | --- | --- | --- | --- | --- | --- | --- | --- | --- | --- | --- | --- | --- | --- |
| **Left Cerebrum** | Size | x | y | z | Size | x | | y | | z | Size | | x | | y | z | |
| Superior frontal G. |  |  |  |  | 1472 | -6 | 18 | | 48 | | |  |  |  | | |  |
| Middle frontal G. | 631 | -32 | 12 | 58 |  |  |  | |  | | |  |  |  | | |  |
| Middle frontal G. | 250 | -42 | 30 | 34 |  |  |  | |  | | | 238 | -52 | 22 | | | 28 |
| Inferior Parietal L. | 613 | -48 | -48 | 48 | 540 | -36 | -52 | | 42 | | | 462 | -48 | -50 | | | 46 |
| Precuneus | 122 | -6 | -60 | 48 |  |  |  | |  | | |  |  |  | | |  |
| Anterior Insula |  |  |  |  | 95 | -32 | 20 | | 2 | | |  |  |  | | |  |
| Caudate body |  |  |  |  | 122 | -16 | 14 | | 6 | | |  |  |  | | |  |
| **Right Cerebrum** |  |  |  |  |  |  |  | |  | | |  |  |  | | |  |
| Superior frontal G. | 937 | 20 | 8 | 70 | 291 | 38 | 44 | | 30 | | | 147 | 24 | 16 | | | 62 |
| Middle frontal G. | 150 | 36 | 32 | 32 | 635 | 26 | 8 | | 60 | | | 187 | 38 | 36 | | | 34 |
| Medial frontal G. |  |  |  |  |  |  |  | |  | | | 113 | 4 | 28 | | | 40 |
| Superior parietal L. |  |  |  |  |  |  |  | |  | | | 543 | 40 | -60 | | | 50 |
| Inferior parietal L. | 1098 | 34 | -46 | 44 | 926 | 40 | -48 | | 46 | | |  |  |  | | |  |
| Precuneus |  |  |  |  | 386 | 8 | -60 | | 48 | | |  |  |  | | |  |
| Anterior Insula |  |  |  |  | 188 | 32 | 24 | | -2 | | |  |  |  | | |  |
| **Left Cerebellum** |  |  |  |  |  |  |  | |  | | |  |  |  | | |  |
| Pyramis |  |  |  |  |  |  |  | |  | | | 121 | -20 | -62 | | | -30 |
| **Right Cerebellum** |  |  |  |  |  |  |  | |  | | |  |  |  | | |  |
| Culmen |  |  |  |  |  |  |  | |  | | | 143 | 34 | -60 | | | -26 |
| Tuber |  |  |  |  | 232 | 34 | -58 | | -30 | | |  |  |  | | |  |
|  |  |  |  |  |  |  |  | |  | | |  |  |  | | |  |

**Table K. Significant regions of activations for direct multiple comparisons during the 2-back working memory paradigm before exercise.**

Using the AFNI program AlphaSim significant clusters were identified for each sub-group prior to participating in the tandem exercise stress test. Control subjects had significant activation patterns within cortical regions involved in normal working memory processes. START subjects activated working memory regions and bilateral cerebellar vermis. The vermis is composed of 9 lobules and START subjects significantly activated the pyramis and culmen. STOPP subjects activated working memory regions, the cerebellar tuber, bi-lateral anterior insula and caudate body. (Size= Cluster Size; G= Gyrus; L= Lobule) (*P<*0.05, AlphaSim).

|  |  | Controls (N=10) | | | | | STOPP (N=18) | | | | | START (N=10) | | | | |
| --- | --- | --- | --- | --- | --- | --- | --- | --- | --- | --- | --- | --- | --- | --- | --- | --- |
| Hemisphere | Location | T | BA | x | y | z | T | BA | x | y | z | T | BA | x | y | z |
| Left | Superior frontal gyrus | 12.15 | 6 | -6 | 8 | 64 | 5.62 | 6 | -15 | 8 | 70 |  |  |  |  |  |
|  | Middle frontal gyrus | 6.62 | 9 | -38 | 46 | 10 | 3.78 | 9 | -46 | 26 | 34 |  |  |  |  |  |
|  | Middle frontal gyrus | 5.60 | 46 | -44 | 44 | -4 |  |  |  |  |  |  |  |  |  |  |
|  | Medial frontal gyrus |  |  |  |  |  | 5.89 | 8 | -6 | 18 | 46 |  |  |  |  |  |
|  | Superior parietal lobule | 4.98 | 7 | -32 | -55 | 52 | 5.54 | 7 | -30 | -56 | 46 |  |  |  |  |  |
|  | Inferior parietal lobule | 7.56 | 40 | -44 | -48 | 56 | 4.11 | 40 | -44 | -48 | 44 |  |  |  |  |  |
|  | Precuneus | 8.48 | 7 | -2 | -62 | 48 |  |  |  |  |  |  |  |  |  |  |
|  | Insula, Anterior |  |  |  |  |  | 5.52 | 13 | -34 | 18 | 2 |  |  |  |  |  |
| Right | Superior frontal gyrus | 9.15 | 9 | 40 | 40 | 32 | 3.92 | 9 | 42 | 38 | 28 |  |  |  |  |  |
|  | Middle frontal gyrus | 9.61 | 6 | 28 | 10 | 48 | 5.38 | 6 | 25 | 15 | 55 |  |  |  |  |  |
|  | Medial frontal gyrus | 9.64 | 10 | 10 | 5 | 50 |  |  |  |  |  |  |  |  |  |  |
|  | Inferior frontal gyrus |  |  |  |  |  | 3.98 | 9 | 44 | 12 | 30 |  |  |  |  |  |
|  | Inferior Parietal Lobule | 4.69 | 40 | 32 | -46 | 38 | 3.99 | 40 | 40 | -48 | 48 |  |  |  |  |  |
|  | Insula, Anterior |  |  |  |  |  | 4.66 | 13 | 30 | 22 | 4 |  |  |  |  |  |
|  | Lateral Globus Pallidus |  |  |  |  |  | 3.65 |  | 12 | 6 | 2 |  |  |  |  |  |
| Left Cerebellum | Anterior Lobe |  |  |  |  |  | 4.26 |  | 0 | -34 | -28 |  |  |  |  |  |
| Right Cerebellum | Anterior Lobe |  |  |  |  |  | 3.81 |  | 12 | -34 | -28 |  |  |  |  |  |
|  | Cerebellar Tonsil |  |  |  |  |  | 6.05 |  | 38 | -54 | -32 |  |  |  |  |  |

**Table L. Functional network for the 2-back WM paradigm after exercise.** Controls and STOPP subjects activated regions associated with working memory In addition STOPP subjects activated regions in the cerebellum. START subjects did not demonstrate any activated regions during the 2-back paradigm. (T=T value; BA=Brodmann's area; Talairach coordinates x, y and z in mm). Whole-brain maps (displayed at *P*< 0.001) were corrected for multiple comparisons at the voxel level at *P*<0.05, FDR.

|  | Controls (N=10) | | | | STOPP (N=18) | | | | START (N=10) | | | |
| --- | --- | --- | --- | --- | --- | --- | --- | --- | --- | --- | --- | --- |
| **Left Cerebrum** | Size | x | y | z | Size | x | y | z | Size | x | y | z |
| Superior frontal G. | 2144 | -6 | 8 | 64 |  |  |  |  |  |  |  |  |
| Middle frontal G. | 419 | -36 | 40 | 36 |  |  |  |  |  |  |  |  |
| Middle frontal G. | 238 | -38 | 46 | 10 | 131 | -46 | 26 | 34 |  |  |  |  |
| Medial frontal G. |  |  |  |  | 2185 | -6 | 18 | 46 |  |  |  |  |
| Inferior frontal G. |  |  |  |  | 367 | -54 | 12 | 10 |  |  |  |  |
| Superior parietal L. |  |  |  |  | 111 | -8 | 66 | 54 |  |  |  |  |
| Inferior parietal L. | 217 | -44 | -48 | 56 |  |  |  |  |  |  |  |  |
| Precuneus | 122 | -2 | -62 | 48 |  |  |  |  |  |  |  |  |
| **Right Cerebrum** |  |  |  |  |  |  |  |  |  |  |  |  |
| Superior frontal G. | 575 | 40 | 40 | 32 |  |  |  |  |  |  |  |  |
| Middle frontal G. |  |  |  |  | 227 | 34 | 30 | 32 |  |  |  |  |
| Medial frontal G. |  |  |  |  |  |  |  |  |  |  |  |  |
| Superior parietal L. |  |  |  |  | 139 | 10 | -64 | 54 |  |  |  |  |
| Inferior parietal L. |  |  |  |  |  |  |  |  |  |  |  |  |
| Precuneus |  |  |  |  |  |  |  |  |  |  |  |  |
| **Right Cerebellum** |  |  |  |  |  |  |  |  |  |  |  |  |
| Tonsil |  |  |  |  | 128 | 38 | -54 | -32 |  |  |  |  |

**Table M. Significant regions activated for direct multiple comparisons during the 2-back working memory paradigm after exercise.**

Using the AFNI program AlphaSim significant clusters were identified for each sub-group during the 2-back paradigm after tandem exercises. Control subjects had significant activation patterns within cortical regions involved in normal working memory processes. STOPP subjects activated working memory regions as well. However, STOPP also had a substantially large activation pattern within the medial frontal gyrus (mPFC) unseen prior to exercise participation. Additional recruitment of significant cerebellar regions of the tonsil was observed. In contrast START subjects did not have any significant regions activated. (Size= Cluster Size; G= Gyrus; L= Lobule) (*P<*0.05, AlphaSim).

| **Group** | **Tract** | **FA value** |
| --- | --- | --- |
| Controls | Right SLF | 0.50 [±0.01] |
| STOPP | Right SLF | 0.50 [±0.01] |
| START | Right SLF | 0.49 [±0.02] |
| Controls | Left SLF | 0.47 [±0.02] |
| STOPP | Left SLF | 0.48 [±0.01] |
| START | Left SLF | 0.46 [±0.02] |

**Table N. Mean Fractional Anisotropy (FA) Values for bilateral Superior Longitudinal Fasciculi.** There was no mean difference between groups. Mean [±95% C.I.].

| Cluster Size | | Corrected *P*-Value | Peak Talairach coordinates (x,y,z) | T-Statistic | Region | R/L |
| --- | --- | --- | --- | --- | --- | --- |
| **Regions of Significant Gray Matter Volume Reduction** | | | | | | |
| **A.** Whole Brain: *Controls volume >START* (dF= 16) | | | | | | |
| 901 | | 0.025 | -14, -91,0 | 5.42 | Lingual Gyrus | L |
|  | | | -15,-91,13 | 4.56 | Cuneus | L |
| **B.** Whole Brain: *Controls volume >STOPP* (dF= 25) | | | | | | |
| 475 | 0.07 | | 29,-54,38 | 4.45 | Superior Parietal Lobule | R |
|  | | | 18,-61,44 | 3.95 | Precuneus | R |
|  |  |  | 26,-60,31 | 3.59 | Precuneus | R |
| **C.** Cerebellum: *STOPP volume > START* (dF= 25) | | | | | | |
| 1411 | | 0.035 | 10,-39,-7 | 3.51 | Culmen | R |
|  | | | 9, -47, -13 | 3.50 | Fastigial Nucleus | R |
|  |  |  | -11,-49,-22 | 3.31 | Dentate | L |
| **D.** Brainstem: *Controls > START* (dF= 16) | | | | | | |
| 130 | | 0.02 | 4, -38,-41 | 3.97 | Medulla | R |
| 58 | | 0.034 | 2,-25,-24 | 3.42 | Pons | R |
| **Regions of Significant White Matter Volume Reduction** | | | | | | |
| **E.** Cerebellum: *STOPP volume > START* (dF= 25) | | | | | | |
| 264 | | 0.012 | -21,-38,-31 | 3.88 | Cerebellar Tonsil | L |
|  | | | -19,-34,-24 | 3.30 | Cerebellar Tonsil | L |
| 82 | | 0.012 | -27,-72,-30 | 3.25 | Pyramis | L |
| **F.** Brainstem: *STOPP volume> START (*dF=25) | | | | | | |
| 231 | | 0.004 | -21,-34,-30 | 3.69 | Pons | L |

**Table O. Significant grey matter and white matter volume reduction using VBM.** Two step non-stationary cluster statistics identified significant differences in cortical regions for (**A)** gray matter volume differences for START compared to controls and (**B)** Trend for gray matter volume differences for STOPP compared to controls. (**C-F)** Subcortical regions such as the brainstem and cerebellum were also identified. R=Right Hemisphere L=Left Hemisphere

| Chronic Multisymptom Illness (CMI) (4)  ≥1 symptom in 2 or 3 categories  Severe vs. mild-moderate | | | Oregon  Gulf War unexplained illness (5) | Kansas-defined Gulf War illness (2)  Moderate/severe | | Chronic fatigue syndrome (CFS) case definition (14)  Fatigue plus 4/8 others | | | | Potential  Consensus  Criteria | |
| --- | --- | --- | --- | --- | --- | --- | --- | --- | --- | --- | --- |
| □  □ | □ fatigue |  | □ fatigue | □  □  □ | □ fatigue / sleep | | □ fatigue | | □ exertional exhaustion (inducible symptoms) | |  |
|  |  |  |  |  |  |  |  |  | □ fatigue | |  |
|  | □ mood / cognition | □ sleep  □ cognitive  □ word finding  □ anxiety  □ depressive  □ moody | □ cognitive / psychological problems |  | □ cognitive / neurological / mood | | □  □  □  □ | □ sleep disturbance  □ memory or concentration | □ cognitive / psychological  □ sleep  □ affect  □ anxiety | |  |
|  | □ myalgia /arthralgia | □ arthralgia  □ stiffness  □ myalgia | □ myalgia / arthralgia |  | □ pain | |  | □ myalgia  □ arthralgia | □ myalgia  □ arthralgia | |  |
| Note extensive exclusion criteria including pregnancy, depression with psychosis, HIV, chronic viral, autoimmune, neoplastic or medical disease. | | | |  |  |  |  |  |  |  |  |
|  |  |  |  |  | □ GI | |  | □ lymph node  □ sore throat | □ nociceptive,  interoceptive & somatosensory  central sensitization | |  |
|  |  |  |  |  |  |  |  | □ headache, migraine |  |  |  |
|  |  |  |  |  | □ respiratory | |  |  |  |  |  |
|  |  |  |  |  | □ skin | |  | □ exertional exhaustion (inducible symptoms) |  |  |  |

**Table P. Case definitions and potential consensus criteria.** Symptoms present > 6 months.

| TN | TP | ∆DBP | SPEC | SENS | *START | *STOPP | SPEC | SENS |
| --- | --- | --- | --- | --- | --- | --- | --- | --- |
| 1 | 33 | 2 | 0.030303 | 1 | 10 | 1 | 0.043478 | 1 |
| 3 | 32 | 6 | 0.090909 | 0.969697 | 10 | 6 | 0.26087 | 1 |
| 5 | 30 | 8 | 0.151515 | 0.909091 | 10 | 5 | 0.217391 | 1 |
| 8 | 28 | 10 | 0.242424 | 0.848485 | 10 | 7 | 0.304348 | 1 |
| 10 | 25 | 11 | 0.30303 | 0.757576 | 9 | 9 | 0.391304 | 0.9 |
| 12 | 23 | 12 | 0.363636 | 0.69697 | 9 | 10 | 0.434783 | 0.9 |
| 13 | 21 | 13 | 0.393939 | 0.636364 | 8 | 11 | 0.478261 | 0.8 |
| 16 | 20 | 14 | 0.484848 | 0.606061 | 8 | 14 | 0.608696 | 0.8 |
| 20 | 17 | 15 | 0.606061 | 0.515152 | 8 | 18 | 0.782609 | 0.8 |
| 21 | 13 | 16 | 0.636364 | 0.393939 | 8 | 19 | 0.826087 | 0.8 |
| ***22*** | ***12*** | ***18*** | ***0.666667*** | ***0.363636*** | ***8*** | ***19*** | ***0.826087*** | ***0.8*** |
| 23 | 11 | 19 | 0.69697 | 0.333333 | 7 | 20 | 0.869565 | 0.7 |
| 27 | 10 | 20 | 0.818182 | 0.30303 | 7 | 22 | 0.956522 | 0.7 |
| 28 | 6 | 21 | 0.848485 | 0.181818 | 5 | 22 | 0.956522 | 0.5 |
| 29 | 5 | 22 | 0.878788 | 0.151515 | 4 | 23 | 1 | 0.4 |
| 30 | 4 | 24 | 0.909091 | 0.121212 | 4 | 23 | 1 | 0.4 |
| 31 | 3 | 25 | 0.939394 | 0.090909 | 3 | 23 | 1 | 0.3 |
| 33 | 2 | 28 | 1 | 0.060606 | 2 | 23 | 1 | 0.2 |

**Table Q.** **Values for receiver operator curve (ROC).**

ROC with postural orthostatic diastolic measurements of CMI subjects (*n*=28) for post-exercise changes established a value of Δ ≥18 mmHg. Patients who met criteria for orthostatic diastolic hypertension had more than one positive diastolic recording after standing. Two or more positive recordings were needed so as to rule out blood pressure cuff error.

*Number of Patients who met the criteria for Orthostatic Diastolic Hypertension by having more than one recorded positive diastolic after standing.

| **Model** | **Fixed** | **Rdm** | **Wt** | **df** | **AIC** | **BIC** | **Log L** | **Test** | **L Rto** |
| --- | --- | --- | --- | --- | --- | --- | --- | --- | --- |
| A1 | GRP +TX +GRP *TX | PT/PD | N | 9 | 7749.1 | 7794.6 | -3865.5 |  |  |
| A2 | GRP + TX | PT/PD | N | 7 | 7754.5 | 7789.9 | -3870.2 | A1 v A2 | 9.3* |
| B1 | GRP +TX +GRP *TX | PT/PD | N | 9 | 7749.1 | 7794.6 | -3865.5 |  |  |
| B2 | GRP +TX +GRP *TX | PT | N | 8 | 8199.1 | 8239.5 | -4091.5 | B1 v B2 | 451.9** |
| B3 | GRP +TX +GRP *TX | NONE | N | 7 | 8364.4 | 8399.7 | -4175.2 | B2 v B3 | 167.3** |
| C1 | GRP +TX +GRP *TX | PT/PD | N | 9 | 7742.7 | 7788.2 | -3862.4 |  |  |
| C2 | GRP +TX +GRP *TX | PT/PD | Y | 14 | 7561.8 | 7632.6 | -3766.9 | C1 v C2 | 190.9** |

**Table R. Model selection for mixed effects statistics.** Sequential model building was employed to select model with best fit. **(A1-2)** First, necessity of interaction was assessed. **(B1-3)** Model for interaction was tested for necessity of random effects. **(C1-2)** Full model with nested random effects was tested for necessity of weights to address heterogeneity of variances. Random (Rdm),L Ratio ( L Rto.), Akaike information criterion (AIC), Bayesian information criterion (BIC), Weighted (Wt), likelihood (L), GROUP (GRP), TREATMENT (TX), PATIENT (PT), PERIOD (PD), no (N), yes (Y). (* *P<*0.009, ***P<*0.0001; Bonferroni corrected).

| **Predictor** | **df** | **Chi-square** |
| --- | --- | --- |
| GRP | 2 | 2.71 |
| TX | 1 | 0.20 |
| GRP*TX | 2 | 9.56* |

**Table S. Type 3 analysis of deviance for change in heart rate by group and treatment.** (**P<*0.036; Bonferroni corrected).

| **Contrast** | **Estimate** | **Std. Error** | **Z** |
| --- | --- | --- | --- |
| 2.1 - 1.1 | 3.48 | 2.34 | 1.49 |
| 3.1 - 1.1 | 3.84 | 2.72 | 1.41 |
| 1.2 - 1.1 | 0.81 | 1.80 | 0.45 |
| 2.2 - 1.1 | 1.89 | 2.14 | 0.88 |
| 3.2 - 1.1 | 9.25 | 2.37 | 3.91* |
| 3.1 - 2.1 | 0.36 | 2.41 | 0.15 |
| 1.2 - 2.1 | -2.67 | 2.01 | -1.33 |
| 2.2 - 2.1 | -1.59 | 1.34 | -1.18 |
| 3.2 - 2.1 | 5.77 | 2.01 | 2.88 |
| 1.2 - 3.1 | -3.03 | 2.44 | -1.24 |
| 2.2 - 3.1 | -1.95 | 2.22 | -0.88 |
| 3.2 - 3.1 | 5.41 | 1.94 | 2.79 |
| 2.2 - 1.2 | 1.08 | 1.78 | 0.61 |
| 3.2 - 1.2 | 8.44 | 2.05 | 4.13** |
| 3.2 - 2.2 | 7.36 | 1.77 | 4.15** |

**Table T. Post-hoc comparisons of heart rate data by patient group and treatment.** 1.1= Controls pre-exercise; 1.2 = Controls post-exercise, 2.1=STOPP pre-exercise, 2.2=STOPP post-exercise, 3.1=START pre-exercise, 3.2 START post-exercise. (* *P<*0.002, ** *P<*0.0006; Bonferroni corrected).
